# Inceptor as a regulator of brain insulin sensitivity

**DOI:** 10.1038/s41598-023-36248-4

**Published:** 2023-07-18

**Authors:** Lisa A. Post, Joshua A. Kulas, Joshua L. Milstein, Sarah V. L. Sebastian, Seyyedmohsen Hosseini-Barkooie, Max E. Stevenson, George S. Bloom, Heather A. Ferris

**Affiliations:** 1 Department of Neuroscience, University of Virginia, Charlottesville, USA; 2 Long-Term Health Education and Training Program, US Army Medical Center of Excellence, San Antonio, USA; 3 Division of Endocrinology and Metabolism, University of Virginia, Charlottesville, USA; 4 Departments of Biology and Cell Biology, University of Virginia, Charlottesville, USA

**Keywords:** Cellular neuroscience, Neuroendocrine diseases, Hormone receptors, Alzheimer's disease

## Abstract

**Abstract:**

While historically viewed as an insulin insensitive organ, it is now accepted that insulin has a role in brain physiology. Changes in brain insulin and IGF1 signaling have been associated with neurological diseases, however the molecular factors regulating brain insulin sensitivity remain uncertain. In this study, we proposed that a recently described protein, termed Inceptor, may play a role in brain insulin and IGF1 resistance. We studied Inceptor in healthy and diseased nervous tissue to understand the distribution of the protein and examine how it may change in states of insulin resistance. We found that Inceptor is in fact present in cerebellum, hippocampus, hypothalamus, and cortex of the brain in neurons, with higher levels in cortex of female compared to male mice. We also confirmed that Inceptor colocalized with IR and IGF1R in brain. We saw little difference in insulin receptor signaling following Inceptor knockdown in neuron cultures, or in Inceptor levels with high-fat diet in mouse or Alzheimer’s disease in mouse or human tissue. These results all provide significant advancements to our understanding of Inceptor in the brain.

**Protocol registration:**

The Stage 1 registered report manuscript was accepted-in-principle on 9 August 2022. This manuscript was registered through Open Science Forum (OSF) on 24 August 2022 and is available here: https://osf.io/9q8sw.

## Introduction

The brain contains an abundance of insulin receptor (IR) and the highly homologous insulin-like growth factor-1 receptor (IGF1R). IR is primarily implicated in glucose metabolism, while IGF1R is important for cellular growth. Both dimeric receptors function by binding to their ligand, either insulin or IGF1, at the surface of the cellular membrane. This results in tyrosine kinase activity and the initiation of an intracellular signaling cascade. There is significant overlap between the signaling cascades of the two receptors and they frequently exist as heterodimers of IR and IGF1R^1^. Cellular resistance to insulin signaling, a prominent feature of type 2 diabetes, has been studied extensively in peripheral tissues. Brain insulin and IGF1 resistance have been described in association with impaired brain function, including Alzheimer’s disease^2–4^. The factors regulating brain insulin and IGF1 resistance remain poorly understood.
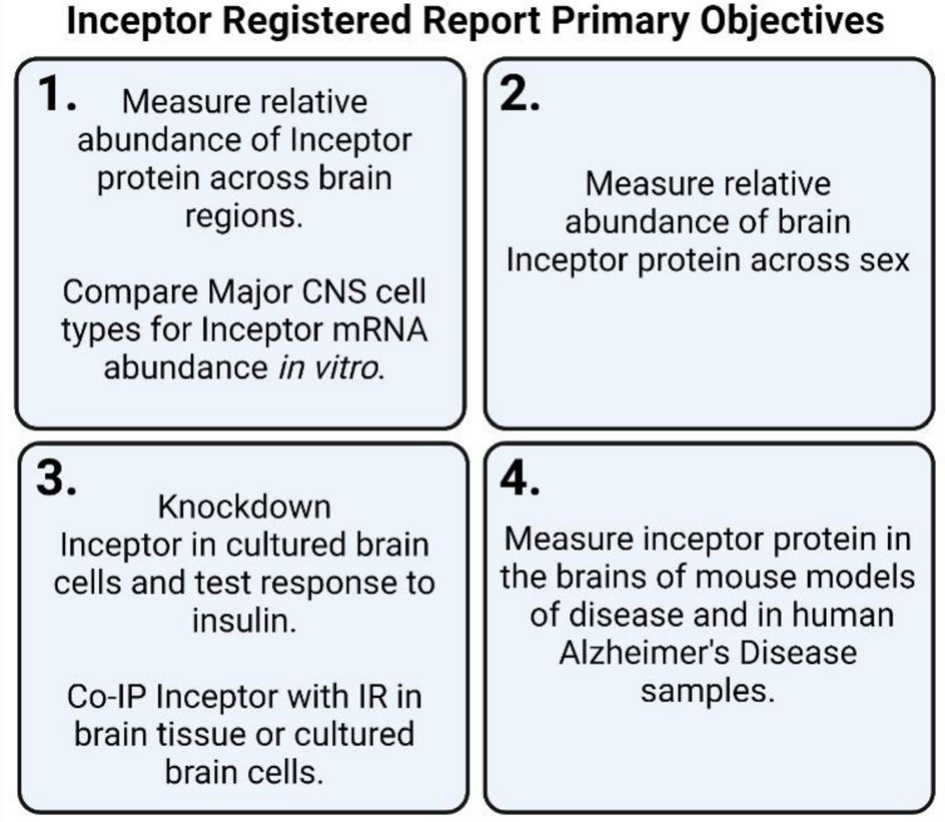


Recent studies have identified a single pass transmembrane protein, termed Inceptor, as a regulator of insulin and IGF1 signaling in the endocrine pancreas^5^. While previous research has shown Inceptor (which has also been called 5330417C22Rik, EIG121, ELAPOR1, and KIAA1324) is present in some cancer cell lines and has effects on autophagy, its role as a regulator of insulin signaling was only recently described^6–9^. Inceptor was found to regulate the insulin sensitivity of pancreatic beta cells by directly interacting with IR and IGF1R to decrease the abundance of these receptors at the cell surface. This resulted in reduced receptor available for cell signaling, but did not impact total receptor levels in the cell. Of further interest, unlike IR and IGFR which are broadly expressed, Inceptor expression appears to be restricted to specific tissues, including pancreas, gastrointestinal tract, and brain. We proposed that Inceptor may play a role in regulating brain insulin and IGF1 signaling. The purpose of this study was to understand the brain region and cell specificity of Inceptor expression in the mouse brain. Moreover, we investigated Inceptor protein differences across sexes, as well as in high-fat diet and Alzheimer’s disease, two models which exhibit brain insulin resistance.

Finally, we compared Inceptor protein levels in autopsy specimens from humans with and without Alzheimer’s disease (Table 1).

**Table 1:** Design Table.

Question	Hypothesis (if applicable)	Sampling plan (e.g. power analysis)	Analysis Plan	Interpretation given to different outcomes
Which brain regions and cell types have the highest Inceptor abundance?	Inceptor protein is more abundant in particular brain regions and cell types	We expected different brain regions and cell types to show differences in Inceptor expression. For tissue, if the true difference in the experimental and control means is 0.4, with a variance of .2, we needed to study 5 experimental subjects to be able to reject the null hypothesis that the population means of the groups are equal with probability (power) 0.8. The Type I error probability associated with this test of this null hypothesis is 0.05. For cell cultures we expected a difference of 0.7, with a variance of 0.2, thus needed 3 cultures per condition. Due to technical limitations (the number of samples possible to fit in a single SDS-PAGE gel), we analyzedn = 3 for each distinct brain region. In the event of an insignificant but clear trend in the data, we would have increased our n by normalizing to Inceptor levels in the hippocampus on each individual blot and pooled this quantitation from multiple blots. Each sample (n) was from an individual mouse. In cell culture experiments, we compared an n of at least 3 unique cultures per cell type. Data was excluded from analysis if it was determined to be a significant outlier by the Grubbs test.	Cortex, hippocampus, hypothalamus, and cerebellum were compared for Inceptor abundance. Inceptor was measured by western blot and normalized to tissue actin. One-way ANOVA with Tukey post hoc test was used to determine significance with *p* < 0.05 considered significant. Astrocytes, neurons, and microglia were compared for *Inceptor* mRNA content. One-way ANOVA with Tukey post hoc test was used to determine significance with *p* < 0.05 considered significant	Inceptor is not significantly changed across regions or Inceptor is not present in some cellular subtypes or brain regions or Inceptor is not expressed in standard CNS-derived primary cell cultures
Is there a sex difference in brain Inceptor expression?	Inceptor expression will differ between male and female brains	We compared the brain regions in which we detected Inceptor protein across sex. We assumed the response within each subject group was normally distributed with standard deviation 0.2. If the true difference in the experimental and control means is 0.4, we needed to study 5 mice of each sex to reject the null hypothesis that the population means of the experimental and control groups are equal with probability (power) 0.8. The Type I error probability associated with this test of this null hypothesis is 0.05	Each brain region had its Inceptor abundance normalized to total Actin. Inceptor/Actin ratio was compared across sex by t-test with *p* < 0.05 considered significant	Females do not have significantly more Inceptor expression than males OR Sex only has effects on particular brain regions
Does Inceptor directly interact with brain IR? Does knockdown of Inceptor in cultured brain cells change cellular response?	Inceptor protein directly interacts with brain insulin receptors and regulates insulin signaling	We compared the effects of insulin stimulation on control and Inceptor knockdown (siRNA treated) cultures. We expected the response within each subject group to be normally distributed with standard deviation 0.2. Based on the report by Ansrullah et al. we predicted a strong effect with a difference in means of 0.5. Thus, we studied 4 control and 4 Inceptor knockdown samples to be able to reject the null hypothesis that the population means of the experimental and control groups are equal with probability (power) 0.8. The Type I error probability associated with this test of this null hypothesis is 0.05	Phosphorylated proteins in the insulin signaling cascade will be normalized to levels of their total proteins. This ratio will be compared by two-way ANOVA with *p* < 0.05 considered significant. This is a change from the registered protocol which in error stated the analysis would be performed by t-test. In co-IP experiments, the result will be binary with either the detection of an Inceptor-IR interaction, or a lack of Inceptor-IR interaction and no statistical analysis will be performed	Inceptor does not interact with brain IR OR Inceptor does not affect cultured brain cell insulin sensitivity OR Inceptor is not present in cultured CNS cells
Do mice with peripheral insulin intolerance or Alzheimer’s disease characteristics have reduced brain Inceptor? and Do human Alzheimer’s brain autopsy samples show changes in Inceptor levels?	Systemic metabolic disruption or Alzheimer’s disease will change Inceptor levels in the brain	We studied a continuous response variable from independent control and experimental subjects with 1 control per experimental subject. Our preliminary data suggested the response within each subject group was normally distributed with standard deviation ~ 0.15. Expecting the true difference in the experimental and control means to be 0.3, we studied 5 experimental subjects and 5 control subjects to be able to reject the null hypothesis that the population means of the experimental and control groups are equal with probability (power) 0.8. The Type I error probability associated with this test of this null hypothesis is 0.05 In experiments of human samples, we utilized frozen control and Alzheimer’s disease patient samples described in the table above. 6 Control frontal cortex and hippocampus samples and 7 AD samples were compared. There is no significant difference in the age of these samples or post mortem interval	Each brain region and pancreas Inceptor abundance was normalized to total Actin. Inceptor/Actin ratio was compared between control and AD mice OR control diet and HFD by t-test with *p* < 0.05 considered significant. Human Inceptor measurements were normalized to total Actin. Inceptor/Actin ratio were compared between control and AD tissue by t-test with *p* < 0.05 considered significant	Peripheral insulin resistance does not change brain and pancreas Inceptor levels or Peripheral insulin resistance changes Inceptor abundance in some brain regions or Only HFD or Alzheimer’s mouse models show changes in brain Inceptor levels or Human AD frontal cortex and/or hippocampus does not have changes in Inceptor protein levels

### Hypothesis 1 – Inceptor protein is more abundant in particular brain regions and cell types

Inceptor protein has been detected in cancer tissues and pancreas. Expression in the hypothalamus has been demonstrated, but its presence in other brain regions has not been characterized^5^. We examined brain tissue for the presence and distribution of Inceptor protein using a commercially-available antibody.

The MIN6 pancreatic insulinoma cell line was used as a positive control in measurements of Inceptor^5^. Tissue lysates were measured for Inceptor level by SDS-PAGE and immunoblot. Our preliminary data supported the performance of this antibody as specific for Inceptor and the presence of Inceptor in the brain, as well as the validity of our controls. Inceptor migrates near its 130 kD predicted molecular weight and is absent in liver and kidney (negative controls) [Supplementary Figure S1a,b]. Mass spectrometry also confirmed that the non-specific band found at approximately ~ 80 kD does not represent Inceptor. Using SDS-PAGE gel slices containing proteins from 150 to ~ 60 kD from MIN6 and mouse liver samples, we found that Inceptor was present in the MIN6 samples as expected, while importantly no Inceptor was found in the mouse liver gel samples [Supplementary Figure S1d,e]. This is further confirmed by qPCR, which showed the presence of Inceptor transcript in MIN6 cells, pancreas and brain and absence in liver and kidney [Supplementary Fig. 1c].

To determine which region of the brain contains the greatest abundance of Inceptor protein, we dissected cortex, hippocampus, cerebellum, and hypothalamus from male and female mice and compared relative Inceptor protein abundance by western blot. We predicted that Inceptor would have a heterogeneous brain distribution and that hypothalamic tissue would have the highest Inceptor expression, due to the hypothalamus’ important role in sensing peripheral insulin and regulating feeding behavior. If Inceptor protein levels were found to be equivalent in all brain regions, this could suggest that Inceptor brain expression is equally important for regulation of the highly-expressed IGF1R and IR.

Our preliminary data demonstrated that Inceptor is present in the brain [Supplementary Figure S1b,c], however, it remained uncertain which CNS (central nervous system) cells express Inceptor. Primary cultures of neurons, astrocytes, and microglia were derived from wild type mice. Mature cultures were collected and relative abundance of *Inceptor* mRNA were measured by quantitative PCR. The housekeeping gene Tata-binding protein (*Tbp*) was used to normalize expression across samples. The MIN6 pancreatic beta cell-derived cell line was used as a positive control in these experiments. In the event we did not detect *Inceptor* in cultured cells, this would suggest that *Inceptor* expression is more abundant in vivo, or not present in these particular CNS cell types. It is also possible that circulating factors which are not present in culture media regulate expression of *Inceptor*. We explored this possibility in greater detail below. Based on available brain RNA sequencing databases, we predicted neurons would have the highest *Inceptor* gene expression^10,11^.

### Hypothesis 2 – Inceptor expression will differ between male and female brains

Previous research identified Inceptor in cancer cells and termed the gene EIG121, or estrogen induced gene 121^9^. Given that estrogen is known to influence Inceptor levels in other contexts, we hypothesized that Inceptor abundance will differ across male and female mouse brains. To test this, 14-week-old C57BL6/J mouse brain tissues were divided into cortex, hippocampus, cerebellum, and hypothalamus. Each brain region from male and female mouse brains was compared directly for Inceptor content by SDS-PAGE immunoblot. In the event we did not detect Inceptor in particular brain regions during the experiments described above, they were excluded from our analysis. We predicted female brains would have higher Inceptor levels than male brains, likely driven by increased exposure to estrogen.

In the event we could not detect changes in brain Inceptor levels across sex, we would have concluded that sex is not a significant factor in regulating brain Inceptor levels. In the event that only some brain regions were affected by sex, this could suggest that estrogen differentially affects these brain regions.

### Hypothesis 3 – Inceptor protein directly interacts with brain insulin receptors and regulates insulin signaling

As stated above, Inceptor has been shown to directly interact with the insulin receptor in the pancreas to regulate insulin signaling. We tested whether this association also occurs in nervous tissue.

To test if Inceptor directly regulates brain tissue insulin sensitivity, we knocked down Inceptor with shRNA introduced via lentivirus particle transduction to the cell type(s) that express it in culture. This experiment was conditional and only proceeded because we were able to detect Inceptor in cultured cells. Following stable uptake of shRNA against Inceptor, cells were treated with 10 or 100 nM insulin and activation of the insulin signaling cascade was measured by western blot.

We tested if Inceptor directly interacts with IR and IGF1R in vivo*.* In an attempt to promote this potential interaction, mice were dosed with 50 uL of recombinant human insulin (Humulin R) into the inferior vena cava 10 min prior to brain tissue harvest. Tissues were collected by dissecting the mouse brain region with the most abundant Inceptor levels and immunoprecipitating IR/IGF1R from the tissue. We then probed for Inceptor interaction by SDS-PAGE/western blot. If a particular sex was determined to have a higher Inceptor expression in the CNS, then that sex was chosen for Co-IP experiments. We predicted that Inceptor would have similar functions in the brain as has been described in the pancreas. We also predicted Inceptor knockdown would increase insulin sensitivity in cultured cells, and more Inceptor would Co-IP with the insulin and IGF1 receptors after insulin stimulation.

### Hypothesis 4 – Systemic metabolic disruption or Alzheimer’s disease will change Inceptor levels in the brain

Studies have shown that prolonged dietary insults can affect the brain adversely. One commonly-utilized metabolic insult is the high fat with high sugar diet (HFD). This diet has been shown to promote both brain and peripheral insulin resistance. We have conducted a 12 week HFD feeding experiment in mice to generate tissue samples which are archived in our laboratory. Following this diet, we performed both glucose tolerance and insulin tolerance tests to confirm the effects of the diet on peripheral metabolic parameters [Supplementary Figure S2]. Our preliminary data demonstrate that these animals are glucose intolerant and insulin resistant. Brains from these mice were collected and flash frozen for biochemical analysis. The levels of Inceptor expression were then measured in cortex, hippocampus, and hypothalamus by western blot as described above. Additionally, little is known about what happens to Inceptor peripherally in insulin-resistant states. To address this, we measured Inceptor levels in pancreas samples from mice fed the HFD versus controls. If Inceptor is driving insulin resistance in response to HFD, we would expect Inceptor levels to be increased. If we did not see an increase, this would suggest that the insulin resistance is primarily driven by previously-described mechanisms^12^.

Brain insulin resistance has been demonstrated in samples of human Alzheimer’s disease (AD) tissue *post mortem*^3^. Our lab maintains the 3xTg mouse model of Alzheimer’s Disease^13^. This model develops significant metabolic perturbations as a function of age^14,15^. We tested 40-week-old female 3xTg AD mice and B6129SF2/J control mice from our colony to confirm these differences [Supplementary Figure S3], and our preliminary data demonstrated that these animals are significantly glucose intolerant and insulin resistant. To confirm that we could detect Inceptor in these aged animals, we measured Inceptor in the hypothalamus (Fig. 4a). We observed a modest, but significant reduction in Inceptor levels in the 3xTg mouse hypothalamus compared to controls. This demonstrated that Inceptor is detectable in the aging mouse brain and that its levels may change in this model as a result of AD neurodegeneration or metabolic impairments. We built off of this observation and measured Inceptor levels in both the cortex and hippocampus of these animals by western blot. We predicted that the hippocampus of 3xTg AD mice would show reduced Inceptor expression as the disease progresses compared to controls. This could suggest that Inceptor is being down-regulated in an attempt to increase IR/IGF1R signaling in the face of other inhibitory signals or that it is being directly impacted by AD pathology.

Finally, to further validate any potential findings in 3xTg animals we measured Inceptor in human Alzheimer’s disease frontal cortex and hippocampus autopsy specimens (see table in Design). We predicted that the hippocampus of human specimens with Alzheimer’s disease would show reduced Inceptor expression compared to controls.

## Methods

### Ethics information

Animal studies were performed in accordance with ARRIVE guidelines and as approved by the Institutional Animal Care and Use Committee at University of Virginia. Human tissue was used as was approved by the Institutional Review Board for Health Sciences Research at the University of Virginia. Samples were obtained with consent from the families of the deceased. All methods were carried out in accordance with relevant guidelines and regulations.

### Design

#### Animals

The C57BL6/J (#000664), B6129SF2/J (#101045), and 3xTg AD (34830-JAX) animals used in this study were purchased from Jackson Labs. Tissues from these animals were flash frozen in liquid nitrogen and stored at − 80 °C.

#### Human Alzheimer’s disease tissue

Human frontal cortex and hippocampus brain samples were previously acquired via generous donation from the University of Virginia Brain Resource Facility and have been continuously stored at − 80 °C since receipt. Portioned human brain samples collected in 1.5 ml Eppendorf tubes were thawed on ice and processed as described in the western blot section below, with the exception that full homogenization sometimes required more than two bullet blender cycles depending on sample size.

As shown in Table 2, a total of 6 patients without pathological evidence of Alzheimer’s disease (mean age 69.5 ± 3.9 years, 67% female, average PMI 10.5 ± 1.8 h) were compared to 7 patients with Alzheimer’s pathology (mean age 74.14 ± 4.9 years, 57% female, average PMI 7.6 ± 1.3 h). There were no significant differences in age (*p* = 0.28) or PMI (*p* = 0.1315) between the groups.

**Table 2:** Human Samples:

Group	Sample	Age	Sex	PMI
AD	223	69	F	11.5
AD	241	73	F	9.5
AD	156	77	F	4.5
AD	178	77	F	4.5
AD	196	66	M	10.5
AD	190	78	M	2
AD	127	79	M	6
Control	228	64	F	17
Control	191	70	F	9
Control	179	72	F	6
Control	216	87	F	13
Control	144	63	M	12
Control	213	61	M	6

#### High fat diet

Mice on a C57BL6/J background were fed a high fat and high sugar diet (Research Diets, cat# D12492i) or control chow (Research Diets, cat#D12450KMi) for 12 weeks. Body weight was recorded each week. At the conclusion of the diet, ITT and GTT testing were performed. The mice were then sacrificed and tissues were harvested and flash frozen.

#### GTT/ITT

For glucose tolerance testing, mice were fasted overnight prior to testing. Mice were weighed and had their baseline blood glucose measured (t = 0) before receiving an IP injection of 10% dextrose at 2 mU/g body weight. Blood glucose was measured by tail clip at 15, 30, 60, 90, and 120 min post-injection. For insulin tolerance testing, mice were fasted for 4 h. Mice were weighed and had their baseline blood glucose measured (t = 0) before receiving an IP injection of Humulin-R insulin at 1 mU/g body weight. Blood glucose levels were measured at 15, 30, 60, 90, and 120 min post-injection by tail clip.

#### Western Blot

Samples were processed by addition of lysis buffer (2.1% SDS, 5% BME, 65 mM Tris–HCl, 0.1% Triton X-100, pH 6.8) containing protease and phosphatase inhibitors on ice. Samples were homogenized using a NextAdvance™ bullet blender for 2 cycles, and then sonicated. Samples were then centrifuged at 14,000×g for 12 min to remove debris. Supernatant was collected and total protein content was measured using the Pierce™ 660 Protein Assay from ThermoFisher Scientific. Samples were prepared for western blot by addition of Laemmli buffer and denatured by 90 °C heat for 5 min before SDS-PAGE and immunoblot. Proteins were resolved using 10–20 ug of protein loaded in 4–16% acrylamide stain-free gels from Biorad. After SDS-PAGE, the gels were imaged for protein in the stain-free gel before transfer. Proteins were then transferred to LF-PVDF membranes and blocked using T20 protein blocking buffer for 1 h. Primary antibodies were diluted at a 1:500 concentration in 5% milk solution and applied overnight at 4 °C. Following primary incubation, membranes were washed 3 times in PBST and secondary antibodies were applied at a 1:4000 concentration in 5% BSA-TBST for 2 h. Membranes were then washed 4 times and imaged. All western blot data was normalized to actin as a loading control. Each western blot sample was a biological replicate from an individual animal.

#### qPCR

The following primer pairs were used in this study; *Inceptor*: F-CACAGGTTCCAGGTGGAGG, R-TGCAAGAGAAGGAGCACTCG. *TBP* F-ACCCTTCACCAATGACTCCTATG, R-TGACTGCAGCAAATCGCTTGG. RNA was isolated from cells using TRIzol extraction and cDNA was synthesized using Applied Biosystems High-Capacity cDNA Reverse Transcription Kit. qPCR was performed using SYBR Green dye.

#### Antibodies

The Inceptor/EIG121 antibody was purchased from Novus Biologicals (NBP2-57699). Actin fluorescent conjugated antibody was purchased from BioRad (4568044). Insulin receptor antibody (3025S), IGF1R antibody (3027S), ERK antibody (9102L), pERK antibody (9106L), and pAKT antibody (9271S) were purchased from Cell Signaling technology. AKT antibody was purchased from Invitrogen (AHO1112).

#### Statistical analysis

Data analysis was performed using GraphPad Prism. T-Tests, one- or two-way ANOVA were performed as appropriate with *p* < 0.05 considered significant.

#### MIN6 beta cell line

The MIN6 cell line utilized in this study was generously donated by Dr. David Castle (University of Virginia). These cells are a positive control for the measurement of Inceptor in this study. These cells were cultured using high glucose DMEM supplemented with 15% FBS, 1% pen strep, and 50uM beta-mercaptoethanol (BME).

#### Neuron primary culture

Primary embryonic neuron cultures were generated using standard methods. Cultures were grown from day 17 embryos. The embryonic cortex was dissected from the brain, mechanically dissociated in ice cold neurobasal media, and then digested in trypsin supplemented with DNAse for 30 min. The digestion was terminated using MEM supplemented with 10% FBS. The cells were pelleted by centrifugation and resuspended in MEM + 10%FBS + Pen Strep and plated on poly-L-lysine-coated plastic 6-well plates. After 2 h, the media was replaced with neurobasal media supplemented with B27 and pen strep. The cells were grown for 12 days before RNA collection or shRNA knockdown.

#### Astrocyte and microglia primary culture

Primary cultures of mixed glia were generated using standard protocols^16^. Day 1-Day 4 neonatal mouse cortical tissue was cleared of meninges and disassociated in trypsin with DNAse for 30 min. Cells were then pelleted by centrifugation. The pellets were then resuspended and seeded in p75 culture flasks for growth. After 1–2 weeks, microglia were separated from the culture using an orbital shaker rotating at 400 RPM for at least 4 h. The microglia were then pelleted from the media to collect RNA. The astrocytes were removed from the culture flask by mild trypsinization and seeded in multi-well cell culture plates for experiments.

#### Co-immunoprecipitation

IR and IGF1R were immunoprecipitated from freshly dissected mouse brain tissue. Anesthetized mice were treated with 5u of recombinant human insulin (Humulin R) 10 min prior to brain tissue collection. The anatomical regions dissected were the regions with the highest Inceptor abundance. Tissue was gently homogenized in RIPA buffer containing protease and phosphatase inhibitors. The homogenate was centrifuged for 20 min at 12,000 RPM and the supernatant was collected. This supernatant was quantified for its protein content. 100–500 µg of lysate was then incubated with IR or IGF1R antibodies overnight at 4 °C. The next day, protein-AG beads were added to the lysate to bind select antibodies for 4 h. Beads were centrifuged to pellet and washed 3 times with lysis buffer. Laemmli buffer was then added to samples for western blot to measure Inceptor.

#### shRNA inceptor knockdown with insulin treatment

We developed 3 lentiviruses encoding various shRNA against *Inceptor,* along with scrambled negative control shRNA, and GFP encoding lentivirus as a positive control to confirm transduction efficiency. shRNA was transduced to cultured cells followed by puromycin selection until stable knockdown of at least 80% of cells was achieved. In the unlikely event that adequate knockdown of *Inceptor* could not be achieved via lentiviral transduction, we would use a morpholino construct to achieve knockdown. Following successful knockdown of Inceptor in the cells, media was replaced with B27 minus insulin for 4 h prior to the start of the experiment. Cells were then left untreated or treated with 10 or 100 nM insulin for 30 min. Cell lysate was collected and activation of insulin signaling was assessed by western blot.

#### Positive and negative controls

The MIN6 pancreatic beta cell line was used as a positive control in measurements of Inceptor (See Supplementary Figure S1 & Fig. 4 for validation). We have validated kidney and liver as negative control tissues (See Supplementary Figure S1).

Mice to be used in this study were assigned to treatment groups based on their sex and genotype. Individual litters were split into multiple treatment groups whenever possible.

Comparisons made in this study were between subjects. Each sample was generated from a unique individual mouse.

Data collection and analysis was not performed blind to the conditions of the experiments.

### Sampling plan

#### Hypothesis 1: Determination of the number of mouse samples in comparisons of brain regions

: We compared different regions of the mouse brain for the presence of Inceptor. We assumed, as in our preliminary data, the response within each subject group was normally distributed with standard deviation 0.15. We expected different brain regions and cell types to show large differences in Inceptor expression. Expecting a true difference in the experimental and control means of 0.3, we studied 5 experimental subjects to be able to reject the null hypothesis that the population means of the groups are equal with probability (power) 0.8. The Type I error probability associated with this test of this null hypothesis is 0.05.

Determination of the number of cell cultures for Inceptor levels by cell type: Based on our preliminary data we expected a > 70% difference in expression between cell types. If the response is normally distributed with a standard deviation of 0.15, we would have needed 2 cultures per group. We expanded this to 3 cultures per group to allow us to assess for outliers.

#### Hypothesis 2: Determination of the number of mouse samples to be used in male versus female experiments

We assumed the response within each subject group was normally distributed with standard deviation 0.2. Based on an expected true difference in the experimental and control means of 0.4, we studied 5 mice of each sex to be able to reject the null hypothesis that the population means of the experimental and control groups are equal with probability (power) 0.8. The Type I error probability associated with this test of this null hypothesis is 0.05.

#### Hypothesis 3: Determination of the number of cell cultures for Inceptor knockdown/insulin signaling experiments

We planned to compare the effects of insulin stimulation on control and Inceptor knockdown (shRNA treated) cultures. We expected the response within each subject group to be normally distributed with standard deviation 0.2. Based on the report by Ansrullah et al.^5^, we predicted a strong effect with a difference in means of 0.5. Thus, we studied 4 control and 4 Inceptor knockdown samples to be able to reject the null hypothesis that the population means of the experimental and control groups are equal with probability (power) 0.8. The Type I error probability associated with this test of this null hypothesis is 0.05.

#### Hypothesis 4: Determination of the number of mouse samples to be used in high fat diet and Alzheimer’s experiments

We planned a study of a continuous response variable from independent control and experimental subjects with 1 control per experimental subject. Our preliminary data suggested the response within each subject group was normally distributed with standard deviation ~ 0.15. Expecting a true difference in the experimental and control means of 0.3, we studied 5 experimental subjects and 5 control subjects to be able to reject the null hypothesis that the population means of the experimental and control groups are equal with probability (power) 0.8. The Type I error probability associated with this test of this null hypothesis is 0.05.

For experiments involving measurements of Inceptor in human AD and control frontal cortex and hippocampus samples, the samples described in the Table of Human Samples (see Design) were used. We expected that human AD samples would have decreased Inceptor abundance in tissue relative to controls.

### Analysis plan

Data was analyzed for statistical significance between groups using GraphPad Prism software. In the event potential outliers were observed in measurements, we performed a Grubbs test to determine if these outliers were significant. Outlier data was thrown out prior to the onset of analysis and were clearly marked in all raw data. Values obtained in experiments were compared using t-test, one or two-way ANOVA with Tukey’s post hoc test where appropriate. A *p*-value of < 0.05 was considered significant and denoted with (*) or the actual *p* value where appropriate.

## Results

### Heterogeneity of Inceptor Levels by Brain Region and Sex

**Figure 1:**
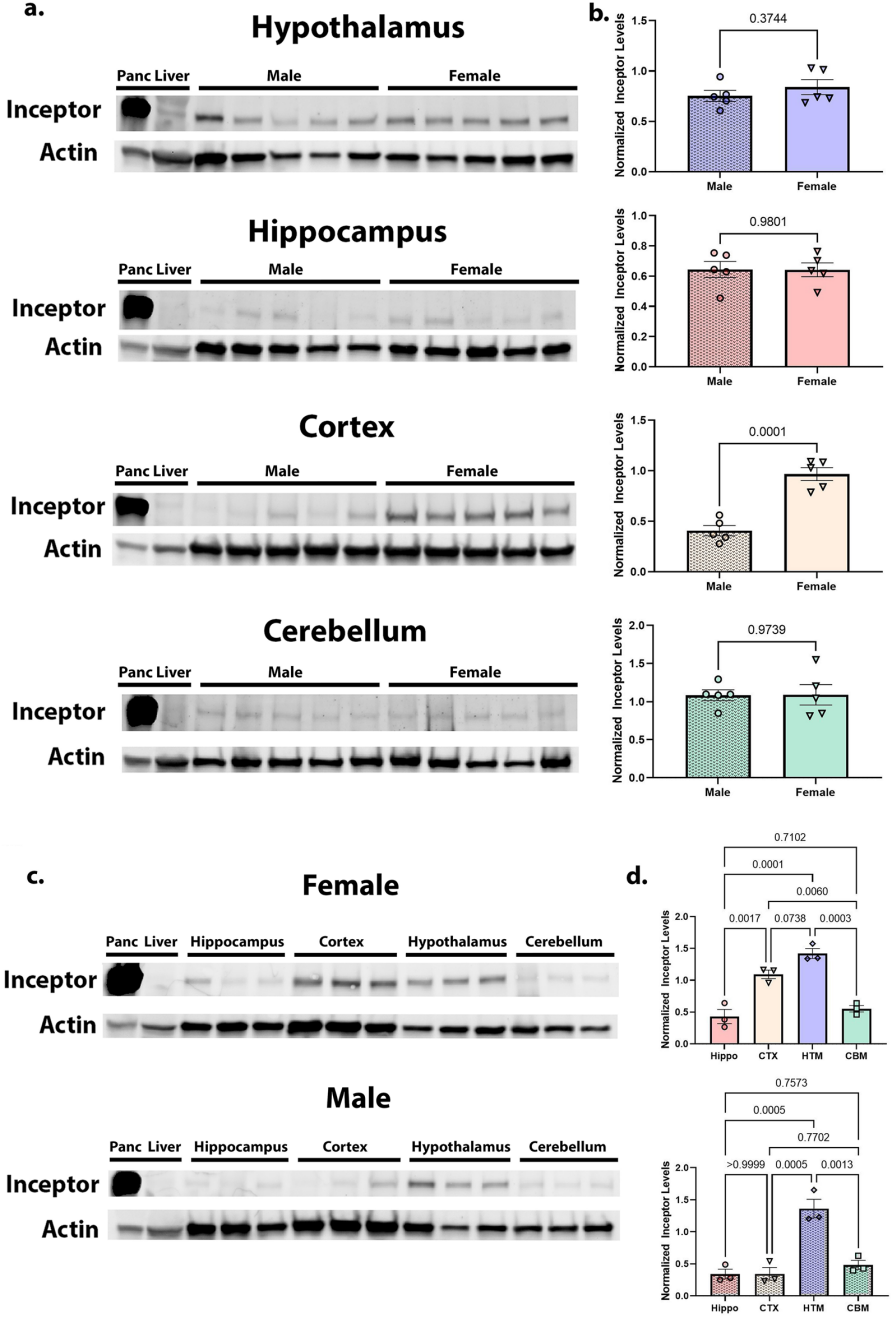
Inceptor levels highest in hypothalamus, female cortex. (**a**) Inceptor levels compared for male versus female 14-week-old C57 mice in hypothalamus, hippocampus, cortex, and cerebellum via western blot. Inceptor protein was normalized to Actin and quantified (**b**). (**c**) Utilizing additional samples from the same 14-week C57BL/6 J mice as in (**a**), relative Inceptor levels were compared across brain regions for each sex via western blot. Inceptor protein was normalized to Actin and quantified (**d**). Statistical significance determined by unpaired t-test (**b**) or one-way ANOVA (**d**).

Following collection of at least 5 male and 5 female 14-week-old C57Bl/6J mice, we prepared tissue lysate from cortex, hippocampus, cerebellum, and hypothalamus for comparison. We also collected pancreas and liver samples from these mice as positive and negative controls, respectively. Pancreas has been demonstrated to be positive for Inceptor [See ref. 5 and Supplementary Figure S1b], and we have also previously shown liver to be negative for Inceptor [Supplementary Figure S1b]. We found measurable levels of Inceptor in all four tested brain regions in both male and female mice [Fig. 1a-b] via SDS-PAGE and western immunoblot. We also found Inceptor distribution to be heterogeneous by both brain region and sex, as we originally hypothesized. In particular, in both males and females, Inceptor levels were lowest in cerebellum and, somewhat surprisingly, hippocampus (Fig. 1c, d). For these two brain regions, male and female levels of Inceptor were equivalent [Fig. 1a, b] (unpaired t-test cerebellum *p* = 0.9739, t = 0.034, df = 8; hippocampus, *p* = 0.9801, t = 0.026, df = 8). Also in accordance with our original prediction, Inceptor levels in the hypothalamus were the highest of any brain region [Fig. 1c, d]. Similar to cerebellum and hippocampus, there were no differences in Inceptor levels by sex in the hypothalamus [Fig. 1a, b] (unpaired t-test *p* = 0.3744, t = 0.941, df = 8).

Notably, in the cortex, we found that Inceptor levels were significantly higher in females as compared to male mice [Fig. 1a, b] (unpaired t-test *p* = 0.0001, t = 6.870, df = 8). In fact, for female mice Inceptor levels in cortex were statistically equivalent to levels in the hypothalamus (one-way ANOVA summary F = 33.98, *p* < 0.0001; by Tukey’s multiple comparisons test cortex vs. hypothalamus adjusted *p* = 0.0738, df = 8), and the Inceptor levels of both these regions were significantly higher than those in cerebellum or hippocampus [Fig. 1c, d] (Tukey’s multiple comparisons test cortex vs. cerebellum *p* = 0.0060, vs. hippocampus *p* = 0.0017; hypothalamus vs. cerebellum *p* = 0.0003, versus hippocampus *p* = 0.0001, all df = 8). For males, hypothalamus showed significantly more Inceptor than all other regions [Fig. 1c-d] (one-way ANOVA summary F = 23.15, *p* = 0.0003; by Tukey’s multiple comparisons test hypothalamus vs. cerebellum *p* = 0.0013, vs. hippocampus *p* = 0.0005, vs. cortex *p* = 0.0005, all df = 8). For all of the data in this section, normality assumptions were met.

### Inceptor is present in neurons

**Figure 2:**
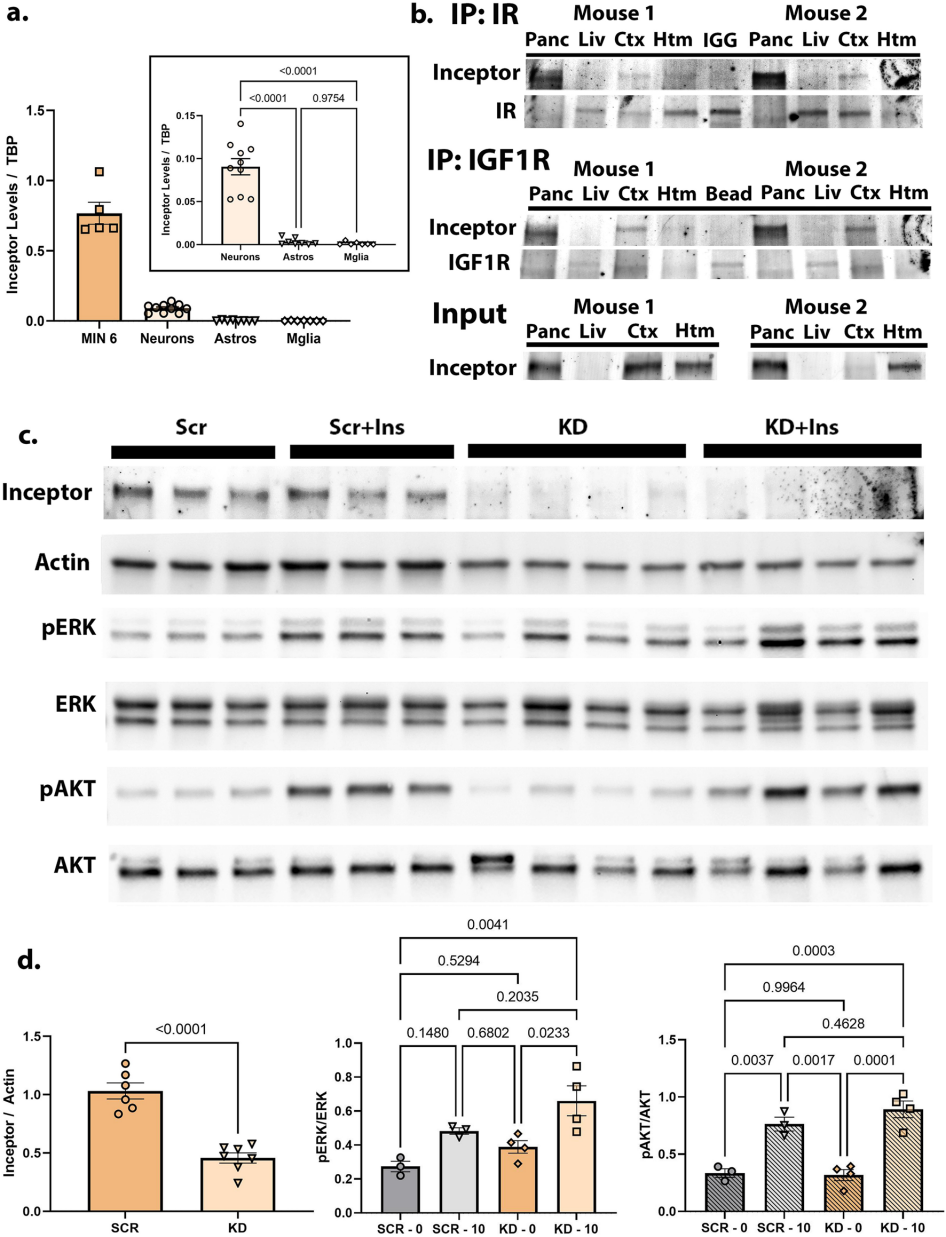
Inceptor is highest in neurons and immunoprecipitated with IR and IGF1R in cortex, but may not significantly change Insulin/IGF signaling following knockdown. (**a**) Inceptor mRNA transcripts measured by cell type using qPCR and quantified relative to tata-binding-protein (TBP) as a control. Cells were cultured from C57BL/6 J mice or MIN6 immortalized line. (**b**) Blots for Inceptor CO-IP, the related IP (IR or IGF1R) plus input sample blots from the pancreas, liver, cortex, and hypothalamus from experiment performed on two animals. (**c**) Knockdown of Inceptor with lentiviral construct #3. Total inceptor knockdown is shown via western blot compared to actin (on one western blot), and a second blot of the same samples shows total levels of pERK, ERK, pAKT, and AKT. Each lane within a group represents a biological replicate (n of 3–4). (**d**) Statistical analysis of western blots. Statistical significance determined with unpaired t-test for Inceptor knockdown, and two-way ANOVA with Tukey for multiple comparisons for pERK/ERK and pAKT/AKT. There was no statistically significant difference in the interaction term (insulin treatment X inceptor knockdown), *p* = 0.1346. Some blots here and in other figures have been contrast and brightness adjusted for maximum visibility. Full raw images of all blots are available as noted in Data Availability section, below.

To determine which cell types in brain have measurable levels of Inceptor, we cultured neurons, astrocytes, and microglia from C57BL/6 J mice, as well as the rat insulinoma cell line MIN6. MIN6 and neuron samples yielded measurable levels of Inceptor via qPCR, while astrocytes and microglia did not [Fig. 2a] (neurons vs. astrocytes and neurons vs. microglia both *p* < 0.0001 by one-way ANOVA with Tukey’s for multiple comparisons). Because astrocyte and microglia data did not meet normality assumptions, we performed a follow-up Kruskal–Wallis test which yielded similar results (neurons vs. astrocytes, *p* = 0.0049; neurons vs. microglia, *p* = 0.0003).

### Co-Immunoprecipitation of Inceptor with Insulin and IGF-1 receptors

We next performed immunoprecipitation (IP) assays to look for interactions between Inceptor and the Insulin receptor or IGF-1 receptor in the brain. We first collected lysate from the pancreas, liver, cortex, and hypothalamus of C57BL/6 J female mice using the protocol as described in Methods. We chose females due to our previous finding that 14-week females had high levels of Inceptor in both the cortex and hypothalamus [Fig. 1]. Since the Inceptor levels of these two brain regions were equivalent, we immunoprecipitated both regions for these tests, as well as pancreas and liver as positive and negative controls, respectively. After immunoprecipitation of Insulin or IGF1 receptors from the lysate according to Methods, we subsequently blotted for Inceptor by SDS-Page and western immunoblot.

For both IR and IGF1R, we successfully co-immunoprecipitated (CO-IP) Inceptor in the pancreas, as had previously been demonstrated by Ansarullah et al.^5^. We found in most cortex samples that Inceptor was also pulled down in both IR and IGF1R IP, but in a few cases Inceptor was not pulled down in the IR IP. We also noted that Inceptor was inconsistently pulled down in the hypothalamus. A representative image of data for one complete animal is provided in Fig. 2a, including the CO-IP for Inceptor, along with Bead or IGG control, the respective IP (IR or IGF1R), and related Input samples. Data from the CO-IP experiments for additional animals are provided in Supplementary Figure S4.

### Insulin / IGF signaling changes following inceptor knockdown

To measure the effects of Inceptor knockdown on Insulin and IGF1 signaling, we next developed three lentiviral constructs and validated their knockdown efficiency compared to GFP and a scramble control [Fig. 2b, Supplementary Figure S5, Supplementary Table 1] (for Fig. 2b, unpaired t-test, *p* < 0.0001, t = 7.264, df = 11). For this knockdown experiment, we utilized neuron cultures since this was the only cell type in the brain with detectable levels of Inceptor in both qPCR and Western blot [Fig. 2a]. Following lentivirus treatment for 3 days, we starved the cells of insulin and then treated with insulin as described in Methods, and collected lysate for western blotting. As markers of Insulin/IGF1 signaling, we looked at levels of both pERK/ERK (also known as pMAPK/MAPK) and pAKT/AKT for the scramble versus knockdown conditions, with and without insulin treatment. Activation levels of pERK/ERK and pAKT/AKT were increased with insulin treatment for both the control and knockdown conditions. Blots from treatment with lentivirus construct 3 and 10 nM insulin treatment are shown in Fig. 2b (by two-way ANOVA, pERK/ERK, interaction term *p* = 0.6068, KD *p* = 0.0312, insulin *p* = 0.0021; for pAKT/AKT, interaction term *p* = 0.2489, KD *p* = 0.3777, insulin *p* < 0.0001. In addition, by Tukey’s multiple comparisons test, pERK/ERK, scr vs. scr + ins *p* = 0.1480; KD vs. KD + ins *p* = 0.0233, scr + ins vs. KD + ins *p* = 0.2035; pAKT/AKT scr vs. scr + ins *p* = 0.0037; KD vs. KD + ins *p* = 0.0001, scr + ins vs. KD + ins *p* = 0.4628; all df = 10). Results for virus constructs 2 and 4 with 10 nM insulin and for all constructs with 100 nM insulin showed similar trends and are provided in Supplementary Figure S5.

For pAKT/AKT, there was a statistically significant difference between insulin treated neurons from the knockdown versus controls groups for lentivirus 4 and 10 nM insulin treatment [Supplementary Figure S5e] (Tukey's multiple comparisons test, *p* = 0.0191, df = 10). However, none of the other lentivirus / insulin treatment combinations showed significant differences between these groups. In some cases, levels of pERK/ERK for insulin-treated cells also appeared to increase even further in the Inceptor knockdown neurons, although this did not reach statistical significance. We note, however, a trend towards significance for lentivirus construct 2 and 10 nM insulin treatment (Tukey's multiple comparisons test, *p* = 0.0604, df = 10, Supplementary Figure S5b). Finally, for the two-way ANOVA, interaction terms (Insulin treatment x Inceptor knockdown) were not significant for any group for ERK or AKT, although for AKT, lentivirus construct 4, 10 nM insulin, *p* = 0.0611 by two-way ANOVA. See full statistics as per Data Availability. For all of the data in this section, normality assumptions were met.

### Inceptor levels unchanged with high-fat diet or in 3xTg mice

**Figure 3:**
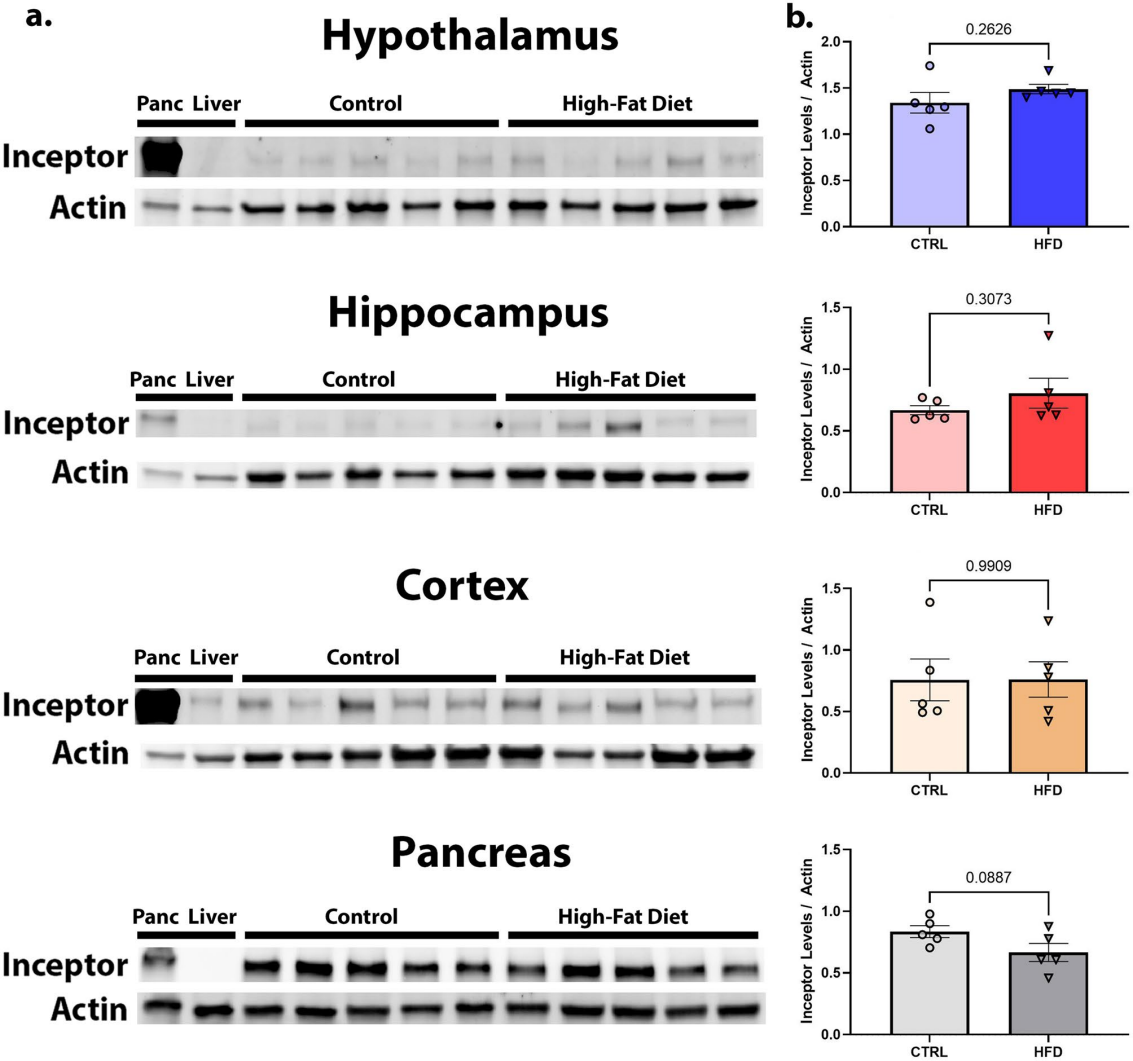
Inceptor levels are unchanged in brain and cortex of HFD mice. Samples from hypothalamus, hippocampus, cortex, and pancreas from 24-week female C57BL/6 J mice previously fed a HFD versus control chow were assessed by western blot for Inceptor levels (**a**) and quantified compared to actin as a loading control (**b**). Unpaired t-test was used to determine statistical significance with *p* < 0.05.

To study the effects of metabolic disturbances on Inceptor, we next tested whether Inceptor levels differed in the brain regions and pancreas of C57BL/6 J mice subjected to a 12-week high-fat and high-sugar diet compared to control animals. We have previously demonstrated that these high-fat diet fed animals are both insulin resistant and glucose intolerant [Supplementary Figure S2]. Utilizing previously collected hippocampus, hypothalamus, cortex, and pancreas samples from female C57BL/6 J mice from this experiment, we found to our surprise that there were no statistical differences in Inceptor levels for any of the brain areas tested by western blotting (unpaired t-test, hypothalamus *p* = 0.2626, t = 1.205; hippocampus *p* = 0.3073, t = 1.090; cortex *p* = 0.9908, t = 0.01173; all df = 8). For hypothalamus, data did not meet assumptions of normality, so we also performed a follow-up Wilcoxon matched-paired signed rank test, which remained not significant with *p* = 0.1250). Similarly, and even more surprisingly, there were no measured differences in the pancreas samples for HFD mice versus controls [Fig. 3a, b], although we note a trend toward a decrease in Inceptor for the HFD mice (unpaired t-test, *p* = 0.0887, t = 1.937, df = 8).

**Figure 4:**
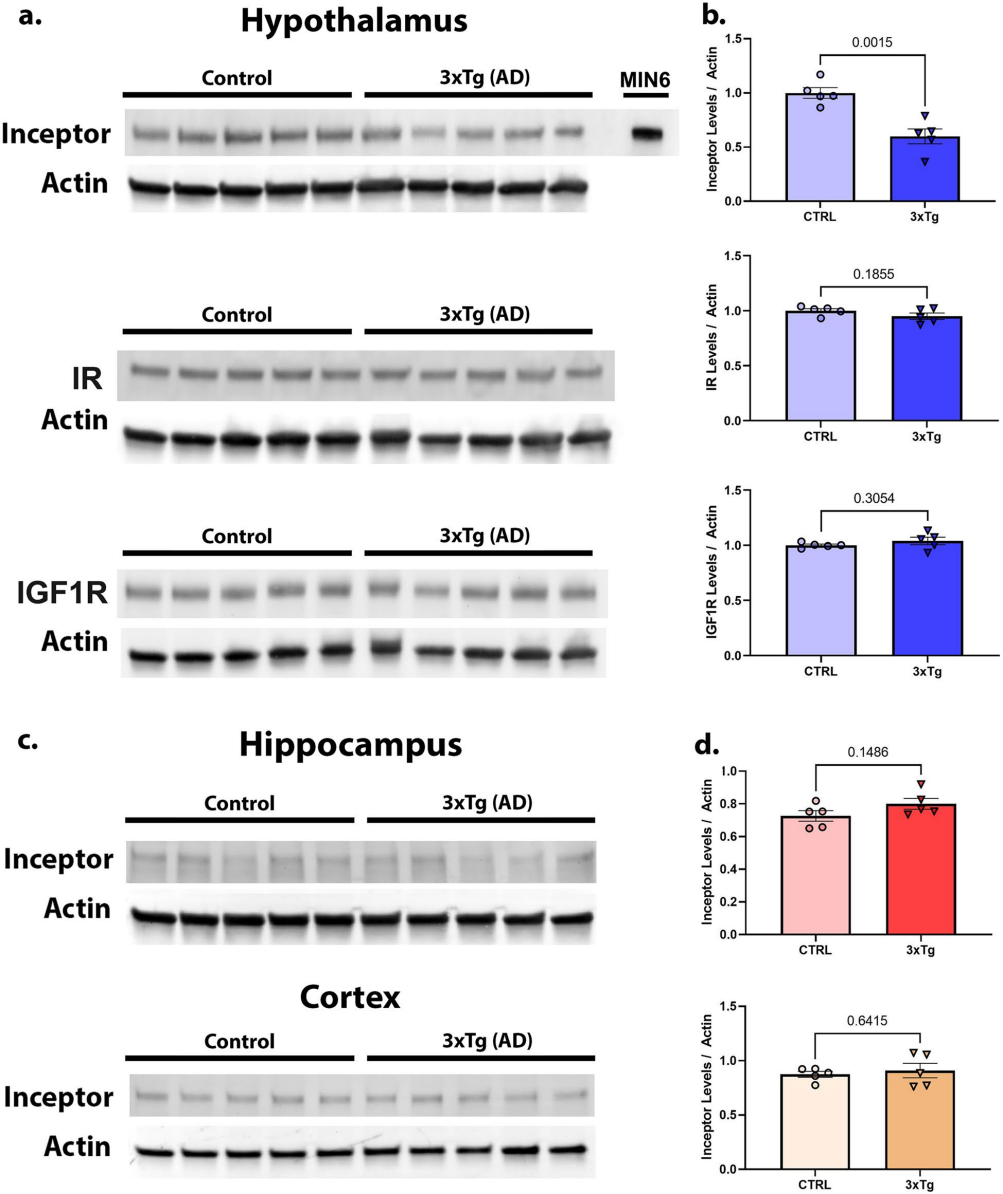
3xTg Inceptor is detectable in the aged mouse brain and reduced in the 3xTg (AD) hypothalamus but not hippocampus or cortex. 40-week-old female mouse hypothalamus tissue was assessed by western blot for Inceptor, Insulin Receptor (IR), and IGF1 Receptor (IGF1R) (**a**), and quantified compared to actin (**b**). Hippocampus and cortex tissue from the same animals was measured for Inceptor (**c**) and quantified compared to actin (**d**). Unpaired t-test was used to determine statistical significance with *p* < 0.05.

We also tested hypothalamus, hippocampus and cortex samples from 40-week old female 3xTg mice versus controls. 3xTg is a commonly used Alzheimer’s disease model utilizing three transgenic insertions which confer both amyloid and tau pathology to the mice, though not neuronal loss^13^. In addition, these mice have been shown to have insulin resistance and glucose intolerance^15^ [Supplemental Figure S3]. In the hypothalamus, where the transgenic insertions are known to express^13–15^ (see Discussion, below), we previously saw that Inceptor levels were significantly decreased for 3xTg mice compared to controls (unpaired t-test, *p* = 0.0015, t = 4.524, df = 8), while total levels of insulin receptor and IGF1 receptor remained unchanged (Fig. 4a, b) (unpaired t-test, IR *p* = 0.1855, t = 1.448, df = 8; IGF1R *p* = 0.3054, df = 8). In contrast, the hippocampus and cortex, which also express the transgenes, showed no such differences in Inceptor for AD mice versus controls (Fig. 4c, d) (unpaired t-test, hippocampus *p* = 0.1486, t = 1.598, df = 8; cortex *p* = 0.6415, t = 0.4837, df = 8). Except as noted above for HFD hypothalamus, for all data in this section, normality assumptions were met.

### Human Alzheimer’s and control brains have similar levels of Inceptor

**Figure 5:**
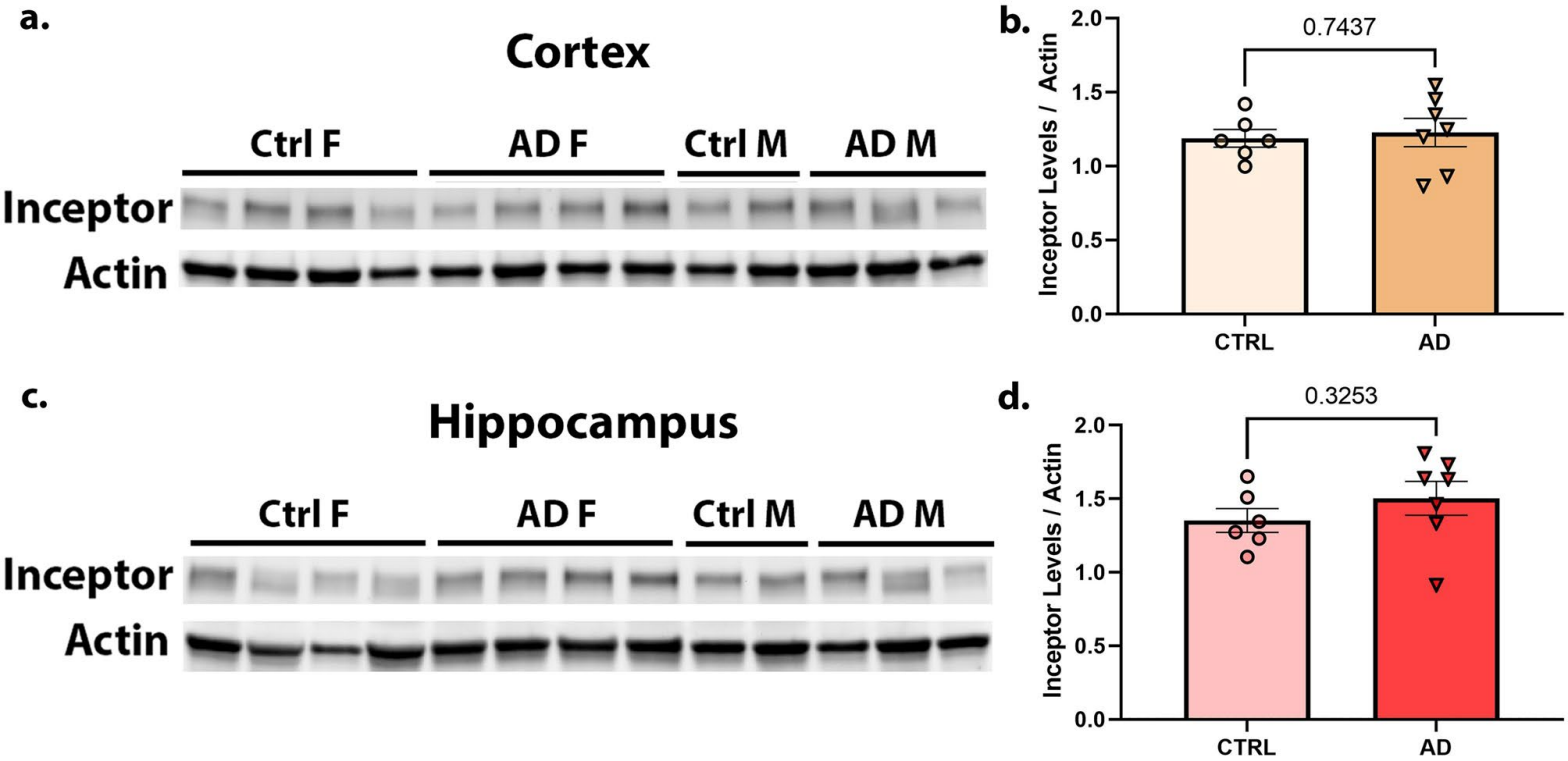
Human Inceptor levels are equivalent in the cortex and hippocampus of AD brain samples versus controls. Male and female cortex (**a**) and hypothalamus (**c**) tissues were assessed by western blot for Inceptor and quantified compared to actin (**b,d**). Unpaired t-test was used to determine statistical significance with *p* < 0.05. Additional data and statistics for these human subjects is provided above in Table 2: Human Samples, in Methods.

Finally, we prepared lysate from human male and female hippocampus and frontal cortex tissue (see Table of Human Samples in Design) from both control and Alzheimer’s disease patients and performed western blotting. Again somewhat unexpectedly, we found no significant differences in the level of Inceptor for AD versus controls for either brain region (Fig. 5a–d) (unpaired t-test, hippocampus, *p* = 0.3253, t = 1.030, df = 11; frontal cortex *p* = 0.7437, t = 0,3353, df = 11). Normality assumptions were met for all human data. This study was not sufficiently powered to examine differences by sex.

## Discussion

The protein labeled herein as Inceptor was initially studied as a prognostic marker in various cancers, as well as for its effects on autophagy and cell survival^6–9^. In 2021, Ansarullah et al. provided evidence of this protein’s presence and actions in the pancreas, aptly dubbing it Inceptor due to its function as an insulin inhibitory receptor^5^. Since then, other groups have continued to study its additional functions, such as in the maturation of normal secretory cells^17^. To our knowledge, however, our work represents the first extensive demonstration in tissue showing Inceptor’s presence in the cortex, hypothalamus, hippocampus, and cerebellum in mice. Our work also provides an initial look into whether these Inceptor levels might vary by sex and disease state, in particular those such as AD and HFD, which are known to have insulin resistance in both the periphery and the brain^1–4^. Finally, our human data provides initial evidence for the presence of Inceptor in the hippocampus and cortex of older human adults both with and without AD.

We noted with great interest the higher levels of Inceptor we found in the cortex of the female C57BL/6 J mice as compared to the males. In light of Inceptor previously being identified as Estrogen-Induced Gene 121 (EIG121) for its upregulation in endometrium in response to estrogen^9^, we did expect to see a sex difference in expression. More interesting was the fact that in the brain, the sex differences were only observed in the cortex. Additional studies delving into the functions and regulatory mechanisms for Inceptor levels in the various regions of the brain could yield interesting results.

As we originally hypothesized, the hypothalamus did indeed have the highest level of Inceptor in both male and female mice in our experiment comparing Inceptor levels by brain region. However, it remains to be seen whether these levels are highest as a result of the hypothalamic role in feeding regulation and peripheral insulin sensing, or for some other reason.

CO-IP experiments showed that Inceptor does likely interact with IR and IGF1R in the cortex, and possibly the hypothalamus as well. Whereas we consistently achieved CO-IP of Inceptor in pancreas samples, the same was not true for the hypothalamus and cortex. One obvious reason is although Inceptor is highest in these brain regions, the levels are low relative to pancreas, which could make it difficult to detect by western blot in some cases. In addition, as nothing is yet known about the kinetics of the activity of Inceptor interaction with IR and IGF1R in the brain, we may miss some interactions. Likewise, a possible explanation is that our insulin treatment of the animals did not adequately stimulate an IGF1R/Inceptor or hybrid receptor/Inceptor interaction, and we are therefore precipitating Inceptor for homeostatic interactions only. Finally, the primary role of Inceptor in the brain may not be to regulate IR and IGF1R. It is possible that there are other receptors or even non-receptor proteins that are regulated by Inceptor in brain.

It came as some surprise that following the knockdown of Inceptor in cultured neurons, the induction of phosphokinases following insulin stimulation remained essentially the same as in cell cultures with intact Inceptor, including at higher insulin treatment doses when both insulin and IGF1 receptors are more completely activated. It is worth noting that insulin and IGF1 receptors are found as either homodimers or a hybrid insulin receptor/IGF1 receptor heterodimer in the cell membrane. It is thought that much of the insulin receptor in the brain is part of these hybrid receptors rather than homodimers. It is unknown if Inceptor will have the same impact on hybrid receptors as homodimers. In addition, there are two isoforms of the insulin receptor, IR-A and IR-B. While neurons contain IR-B, pancreatic beta-cells contain both IR-A and IR-B^1,18^. It is possible that there may be differences in Inceptor influence based on which insulin receptor isoform is present. Altogether, these initial experiments leave many unanswered questions about how Inceptor might regulate insulin/IGF1 signaling and might itself be regulated in the brain.

Previous research in pancreatic neuroendocrine tumors found that higher levels of Inceptor are correlated with better prognosis and outcomes^19^. And as has been shown previously (Ref.^5^), we saw robust levels of Inceptor in our pancreas samples from mice. However, there was no measurable difference in Inceptor levels in the pancreas of mice with a high-fat diet induced insulin resistant state compared to controls, which was somewhat unexpected as these mice have body-wide insulin resistance and metabolic abnormalities. Since we did see a trend towards decreased Inceptor in pancreas from these HFD mice, this suggests that our sample size might be too small to detect subtle differences. The direction of effect also differs from our hypothesis that these levels would increase, and suggests that any differences in Inceptor levels are an effect and not a driver of this resistance.

In the AD model mice, which have transgene-induced metabolic abnormalities and insulin resistance in addition to their neurodegenerative disease phenotypes, only the hypothalamus showed any difference in Inceptor levels between transgenic animals and controls. We note that due to the specific transgenics of this model there is deposition of amyloid and tau in the hypothalamus, a phenotype not replicated in humans^13–15^. Therefore, more important is the fact that the cortex and hippocampus, areas more relevant to human AD, show no differences in Inceptor levels. This suggests that the insulin resistance seen in AD brains is a result of mechanisms not related to Inceptor. Our human data, wherein we also saw no significant differences in Inceptor levels for hippocampus and cortex samples from subjects with AD versus control brains, suggest the same thing.

Our study provides initial evidence for the presence of Inceptor in the brain with variability by sex, brain region, and cell type. We further showed that Inceptor interacts with IR and IGF1R to perform its functions. Further experiments showed that at least in our experimental models, downstream insulin signaling markers did not greatly differ following knockdown of Inceptor, and that Inceptor levels do not significantly vary following high fat diet in mice, or in AD in both mice and humans. These results further highlight that much more remains to be learned regarding the kinetics and mechanisms of Inceptor interactions and regulation, as well as the differences between Inceptor action in the brain versus the periphery.

## Supplementary Information

Supplementary Information.

## Data Availability

All raw data associated with this study has been uploaded to Mendeley Data and is available here: https://data.mendeley.com/datasets/mpt3cwd8rh/1.

## Code availability

This study does not involve the use of coding.
