# Supplementary material for: Inceptor as a regulator of brain insulin sensitivity
Source: Sci Rep. 2023 Jul 18;13:11582. doi: 10.1038/s41598-023-36248-4 (PMC10354159; doi:10.1038/s41598-023-36248-4)

Supplementary Information/Preliminary Data

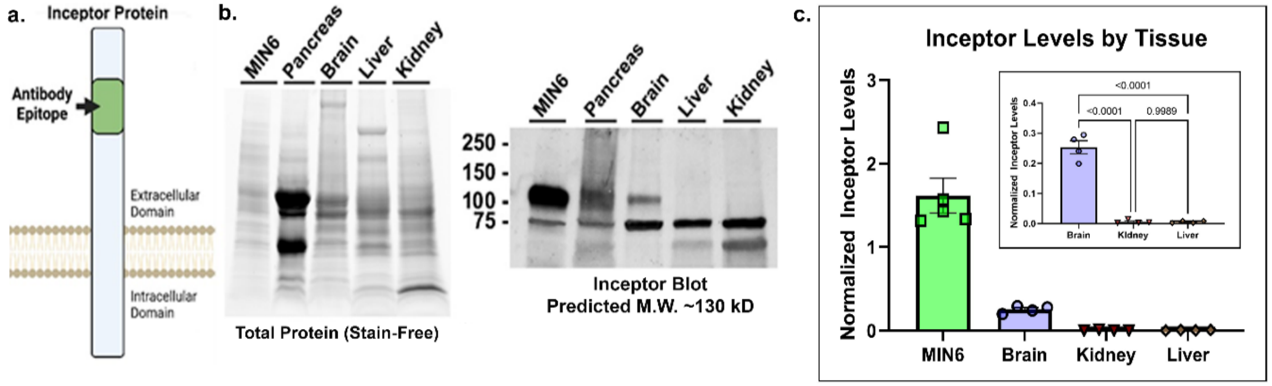

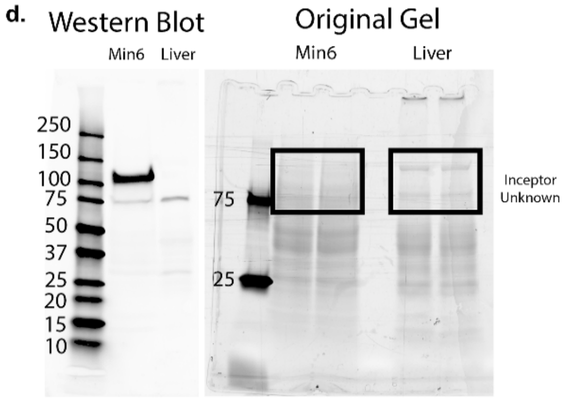

e.

Inceptor Fragment Sequence	Min6 Score	Liver Score
K.ESEYHFEYTACDSTGSR.W	442.5	nf
R.VAVPHSPGLCTSLPDPVK.G	692.6	nf
R.TTAFSVWSK.V	345.6	nf
R.NIAITGVAYTSECFPCKPGTYAAK.Q	846.1	nf
K.LCPANYYSNK.G	439.7	nf
R.DSGTCHLCPSNTILK.A	397.1	nf
K.YFHHFTLSLCGNQGK.K	404.5	nf
R.IPDGEAGFSK.S	370.8	nf
K.SVTAYVCQVVIIPSEVMGYK.A	424.8	nf
K.LCSGGISLPEQR.V	528.5	nf
K.TIDFWLK.V	353.3	nf
K.LVMNATLK.D	387.3	nf

Figure S1. Validation of an Inceptor antibody and detection of Inceptor protein and transcript in tissue lysate. (a) The model displays Inceptor as a single pass membrane domain. The extracellular epitope of Inceptor targeted by the antibody utilized in this study is shown. 10-week-old female C57BL/6J mouse tissue was processed to examine Inceptor (b) protein levels and (c) transcript levels. MIN6 pancreatic beta cells and whole pancreas lysate were used as positive controls, while liver and kidney tissue served as negative controls. TATA-binding protein was used to normalize qPCR data. (d) MIN6 and liver samples (boxes) were cut out following gel electrophoresis, guided by western blotting of duplicate samples on left, to include both the anticipated Inceptor band (~130kD) and the currently unknown band (~80kD). (e) Gel sample proteins were interrogated by mass spectrometry (see supplementary methods). MIN6 samples contained Inceptor protein fragments, whereas no Inceptor was found in the liver samples containing the unknown band. Sequences with scores over 300 were considered of sufficient quality. nf = not found. See Supplementary Datasets 1 and 2 for full list of proteins identified in Min6 and liver samples.

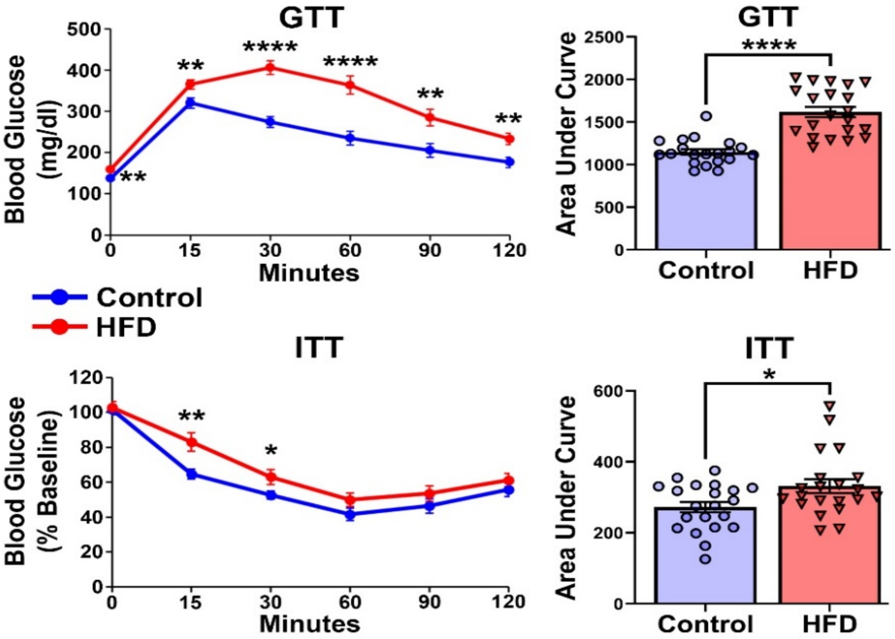

Figure S2. High fat and high sugar-fed mice are glucose intolerant and insulin resistant.
Male and female mice on a C57BL/6J background were fed either a control diet or a high fat and glucose diet for 12 weeks. Animals were then tested for glucose tolerance and insulin resistance. Unpaired t-test was used to determine statistical significance with p<0.05*; p<0.01**; p<0.001***; p<0.0001****.

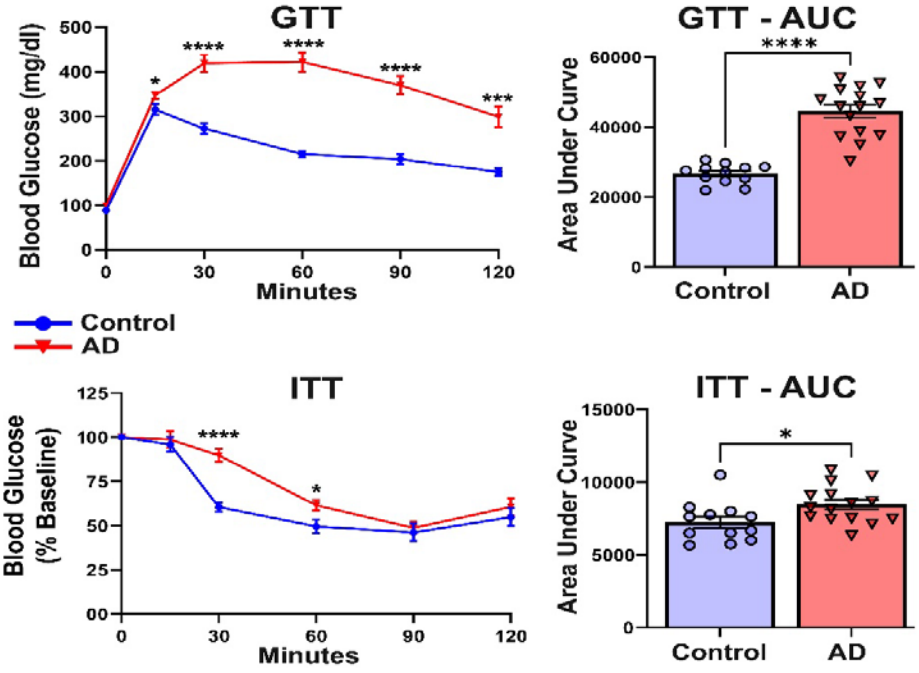

Figure S3. 3xTg Alzheimer's disease mice are glucose intolerant and insulin resistant.
40-week-old female mice B6129SF2/J control mice and 3xTg AD mice on a normal chow diet were tested for glucose tolerance and insulin resistance. Unpaired t-test was used to determine statistical significance with p<0.05*; p<0.01**; p<0.001***; p<0.0001****.

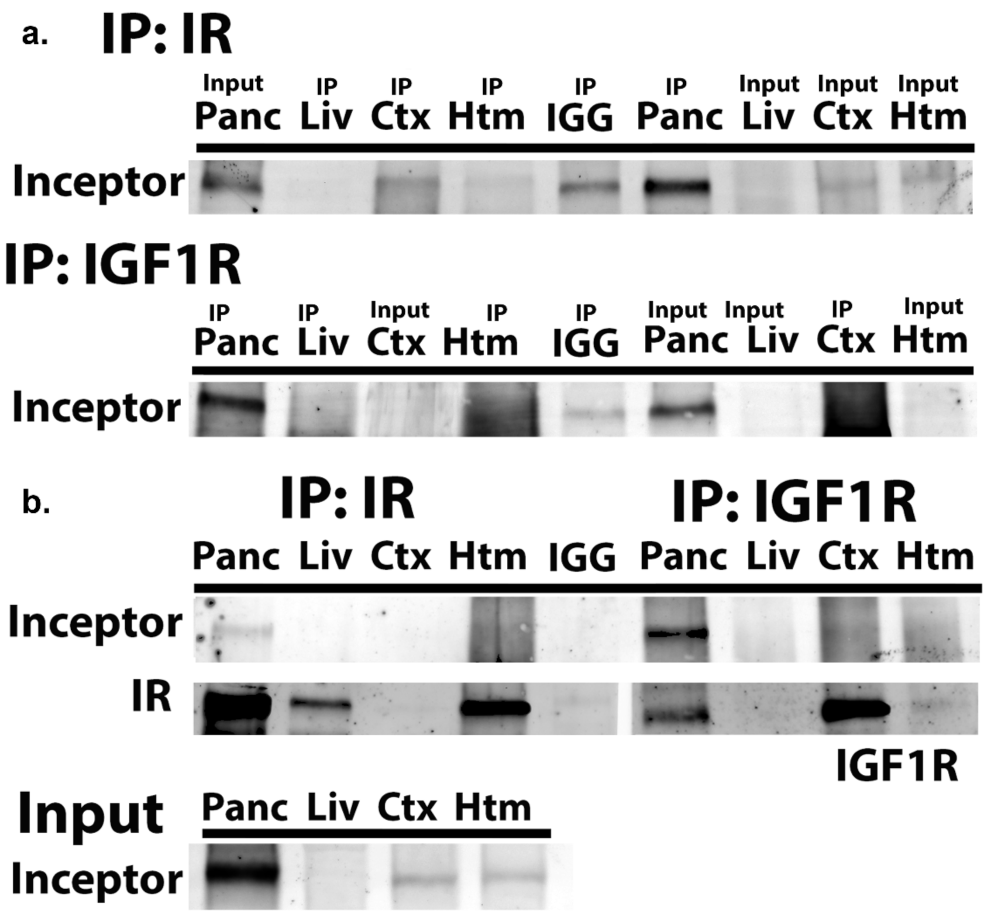

Figure S4. Inceptor can Co-immunoprecipitate with IR and IGF1R in cortex and/or hypothalamus. (a) and (b), each display CO-IP data for an individual mouse, as labeled. Note that for (a), IR and IGF1R blots were not available, and the input and IP samples are combined on one blot as shown. Misloading / exchange of input and IP samples for the pancreas (IR-IP) and Cortex (IGF1R-IP) is also labeled. In (b), the IR and IGF1R CO-IP data from one mouse are on a single blot for Inceptor as indicated, which was then cut and separated for subsequent IR or IGF1R blotting.

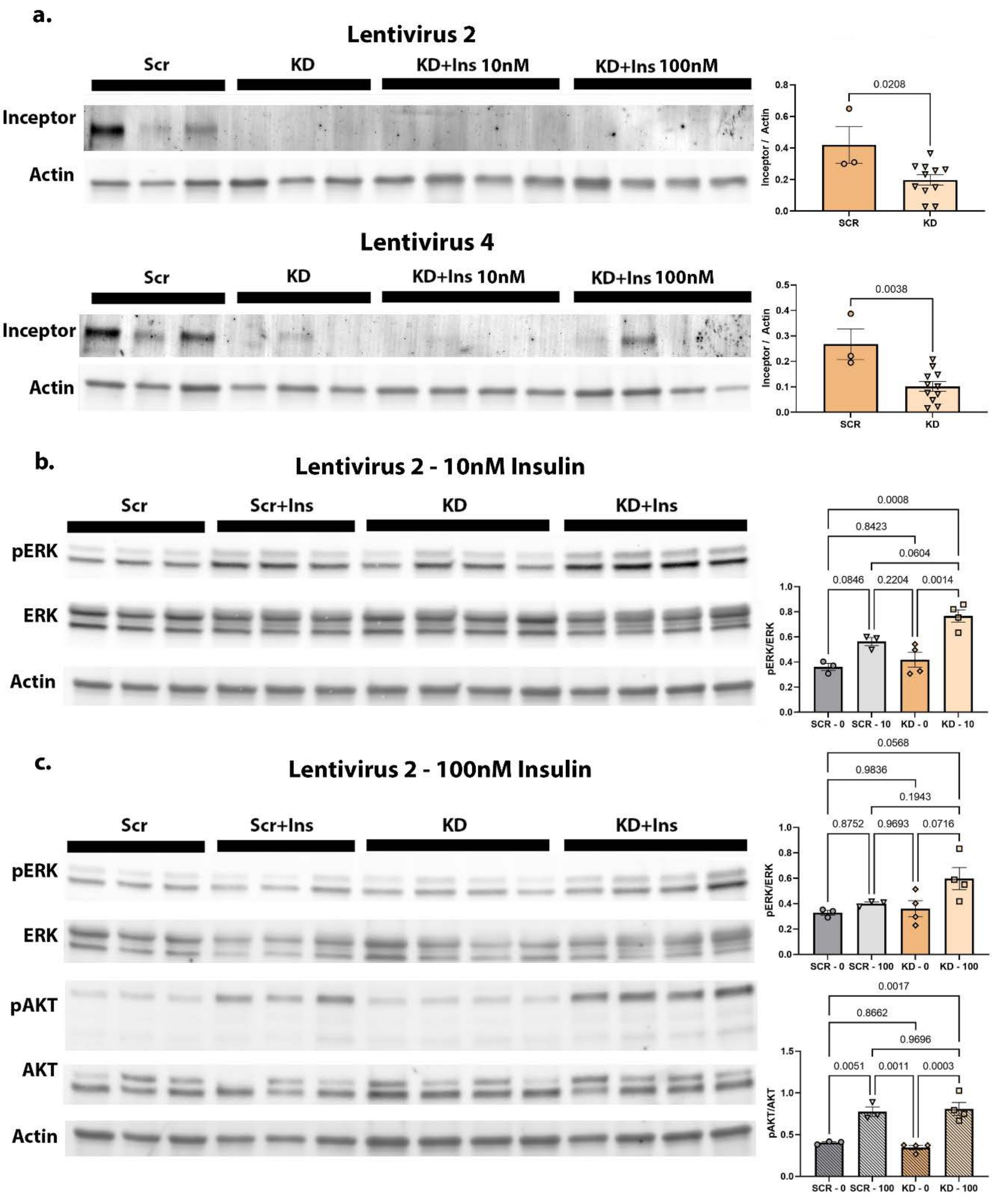
a.
Lentivirus 2
Scr
KD
KD+Ins 10nM
KD+Ins 100nM
Inceptor
Actin
Inceptor / Actin
0.0208
SCR
KD
Lentivirus 4
Scr
KD
KD+Ins 10nM
KD+Ins 100nM
Inceptor
Actin
Inceptor / Actin
0.0038
SCR
KD
b.
Lentivirus 2 - 10nM Insulin
Scr
Scr+Ins
KD
KD+Ins
pERK
ERK
Actin
pERK/ERK
0.0008
0.8423
0.0604
0.0846
0.2204
0.0014
SCR - 0
SCR - 10
KD - 0
KD - 10
c.
Lentivirus 2 - 100nM Insulin
Scr
Scr+Ins
KD
KD+Ins
pERK
ERK
pAKT
AKT
Actin
pERK/ERK
0.0568
0.9836
0.1943
0.8752
0.9693
0.0716
SCR - 0
SCR - 100
KD - 0
KD - 100
pAKT/AKT
0.0017
0.8662
0.9696
0.0051
0.0011
0.0003
SCR - 0
SCR - 100
KD - 0
KD - 100

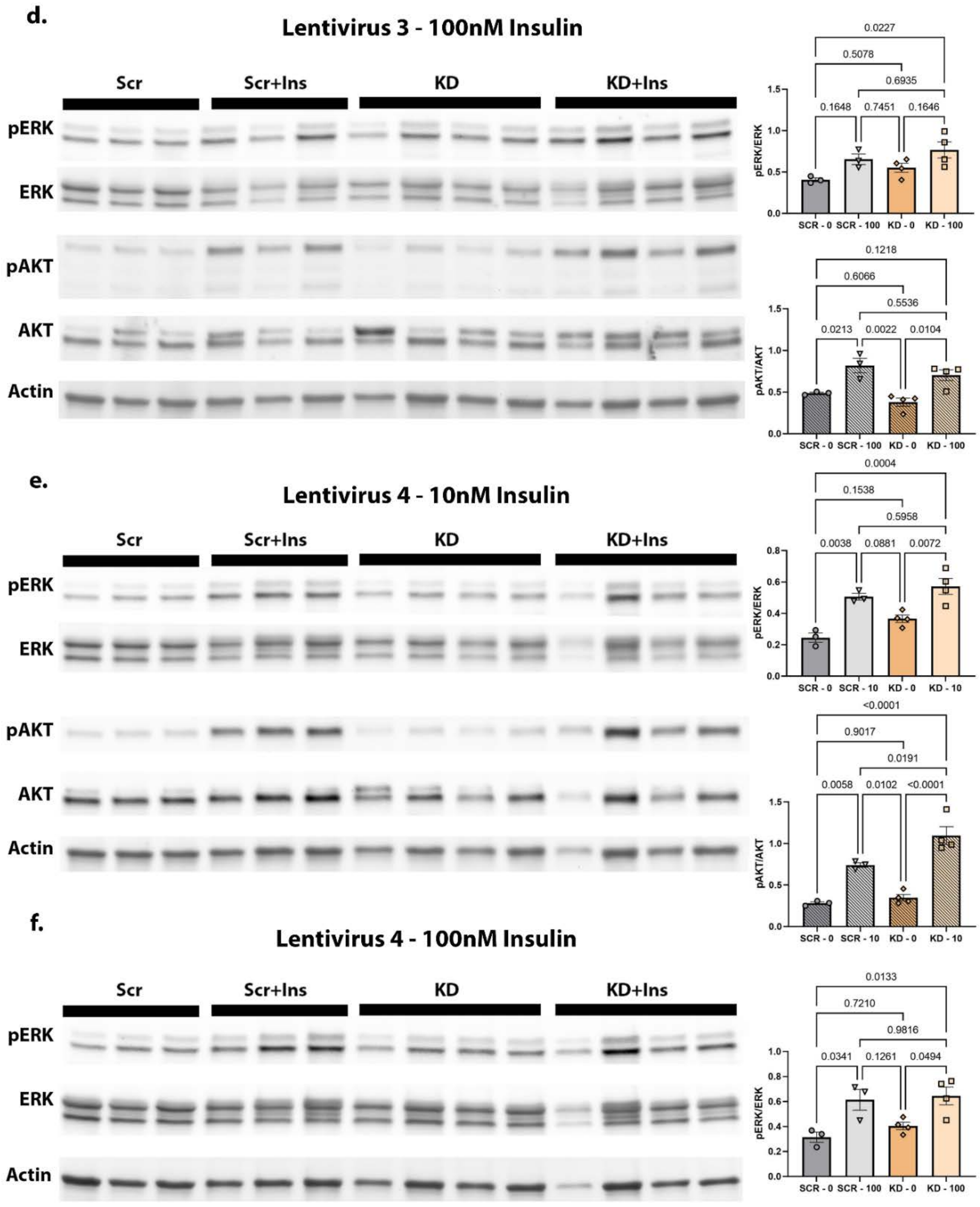

Figure S5, Inceptor knockdown may increase insulin signaling activation. (a) Inceptor knockdown for two additional lentivirus constructs. (b – f) pERK/ERK, actin, and pAKT/AKT data for control versus Inceptor KD neurons. Each lane within a group represents a biological replicate, 3 of the same 4 scramble constructs repeated for each blot (total n of 4 each for SCR, SCR + 10nM Insulin, and SCR + 100nM Insulin). Statistical significance determined with unpaired t-test for Inceptor knockdown, and two-way Anova with Tukey for multiple comparisons for pERK/ERK and pAKT/AKT. No statistically significant difference in any interaction term (insulin treatment X inceptor knockdown). For details, see 'Data Availability'.

Supplementary Table 1. Lentivirus Constructs

Lentivirus	Sequence	Origin / Development	Disposition
**1**	**CAGTGGGATCAACTTTGAATA**	Horizon Discovery Si Smart Pool	Rejected / No KD
**2**	**GGGATCCAGAAGACTACTTAC**	Sigma Aldrich VectorBuilder Library	Success / KD achieved
**3**	**CAGCCTTGCTGATCGACTTAT**	Sigma Aldrich VectorBuilder Library	Success / KD achieved
**4**	**AAAGCATACAACTTTGTGACT**	Whitehead Institute	Success / KD achieved

Supplementary Methods

Sample preparation and mass spectrometry (MS) analysis. Gel pieces were rinsed twice with pure water, and twice with 50 mM bicarbonate/ 50% acetonitrile (ACN) solution. 10 mM dithiothreitol (DTT) was added and the gel pieces were reduced for 30 minutes at 55 °C, following which the solution was discarded. 55 mM iodoacetamide (IAA) was then added, and the mixture incubated at room temperature for 30 minutes in the dark to allow alkylation. IAA was removed and the gel fragment was soaked in ACN. Gel pieces were washed twice with 50 mM bicarbonate solution followed by ACN washes, and finally residual ACN was removed using speed vacuum for 5 minutes.

To digest, 1.5 microgram of trypsin was added, and protein digestion was performed overnight in 50 mM bicarbonate solution at 400 rpm at 37°C. The next day, resulting peptides were collected in low bind tubes. The gel pieces were soaked in 30%ACN/3% Trifluoroacetic acid (TFA) solution to collect all peptides. Finally, the gel fragment was soaked in ACN. Collected solutions from the gel were mixed, frozen and ACN was removed using speed vacuum for 1 hour. To prepare the samples for MS analysis, the stage tip protocol was carried out, and the resulting peptides were solubilized in mobile A for mass spectrometry analysis (HPLC water/ 0.1% Formic acid).

For identification of purified peptides, a data dependent method (top 10) was carried out over 180 minutes as previously described[1]. For identification of the peptides, acquired MS files were searched based on the following parameters using Byonic software: specific trypsin cleavage after lysine and arginine, max 2 missed cleavages, 10 and 50 ppm mass tolerances for precursor and resulted fragments, respectively, carbamiodomethyl as fixed modification at cysteine residues (+57.021464) and oxidation as a common modification at methionine residues (+15.994915).

Supplementary Dataset 1 – MIN6 Mass Spectrometry Data: Spreadsheet containing summary data and full list of proteins identified by Mass Spectrometry analysis in MIN6 cell sample. In this analysis, Inceptor is identified as: A2AFS3|ELAP1_MOUSE Endosome/lysosome-associated apoptosis and autophagy regulator 1.

Supplementary Dataset 2 – Mouse Liver Mass Spectrometry Data: Spreadsheet containing summary data and full list of proteins identified by Mass Spectrometry analysis of mouse liver sample from C57BL/6J mouse. In this analysis, Inceptor (ELAP1_MOUSE Endosome/lysosome-associated apoptosis and autophagy regulator 1) is not found.

Supplemental Bibliography

1. Hahm, H. S. *et al.* Global targeting of functional tyrosines using sulfur triazole exchange chemistry. *Nat Chem Biol* **16**, 150 (2020).

Full Western Blot Images

Blots from Figure 1a

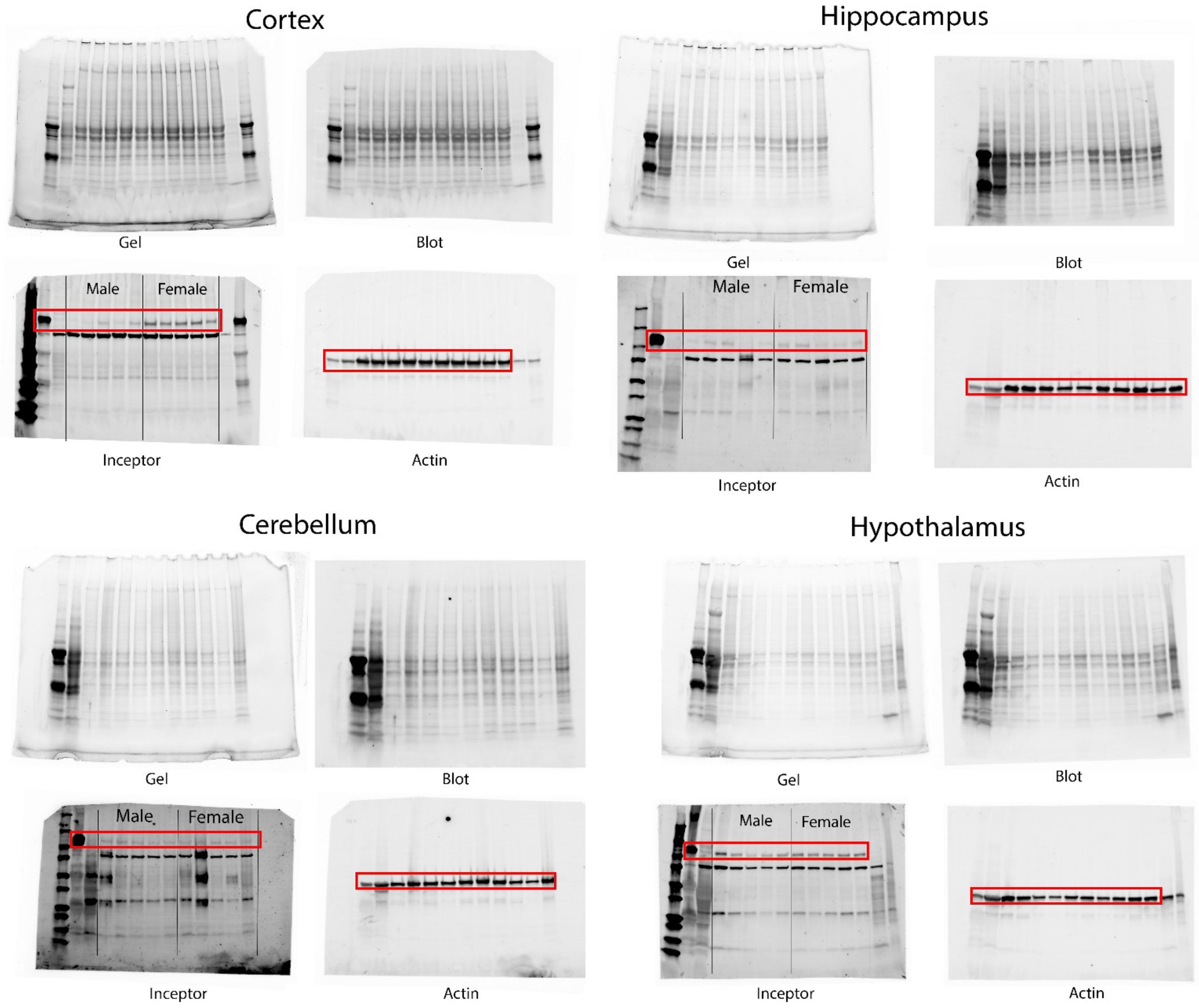

Blots from Figure 1b

Inceptor by Brain Region - Female

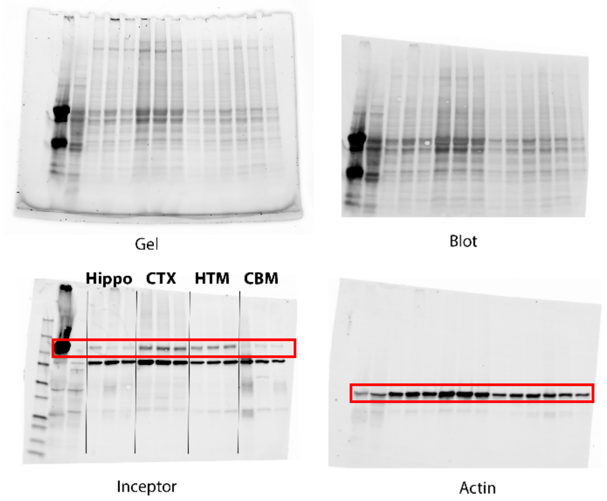

Inceptor by Brain Region - Male

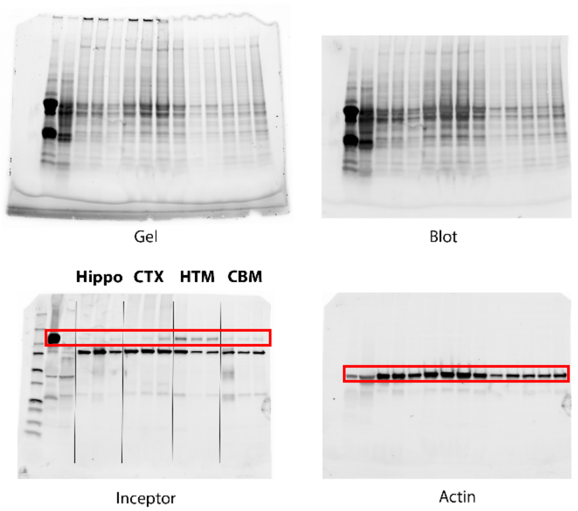

Blots from Figure 2b – CO-IP blots

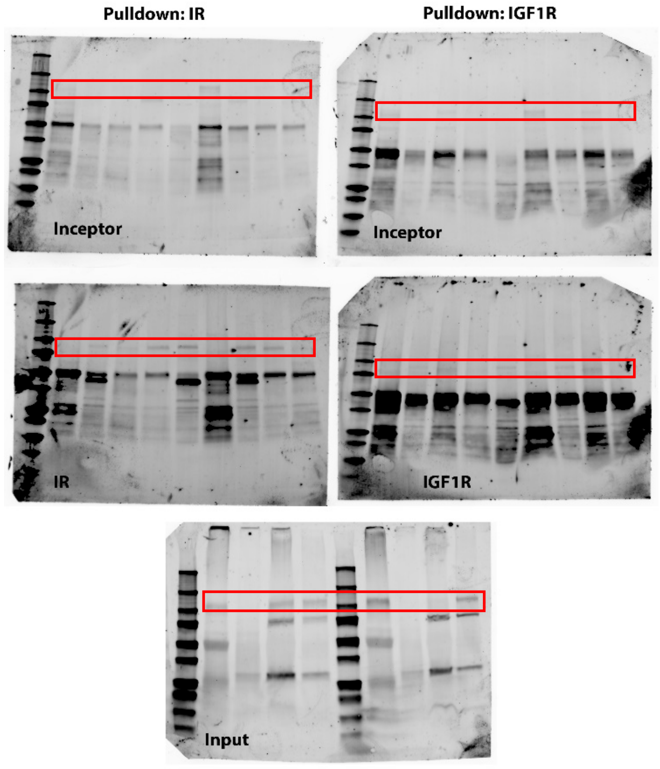

Blots from Figure 2c (Lentivirus 3, 10nM) and Figure S5 (Lentivirus 2, 3-100nM and 4)

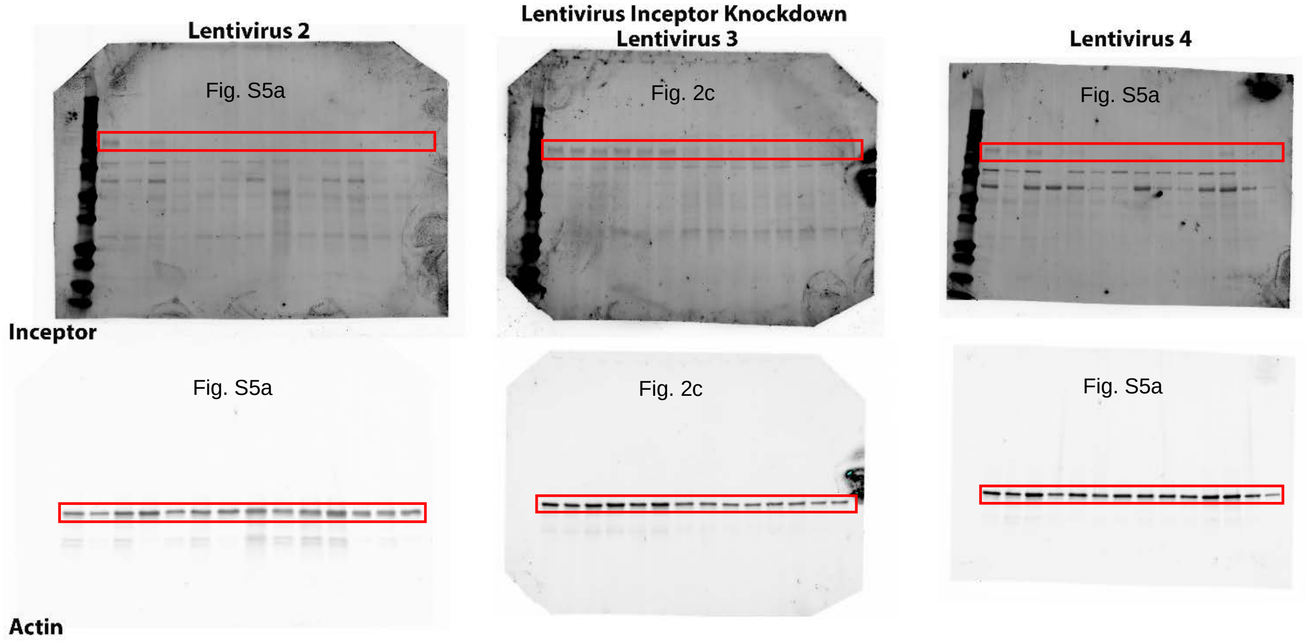

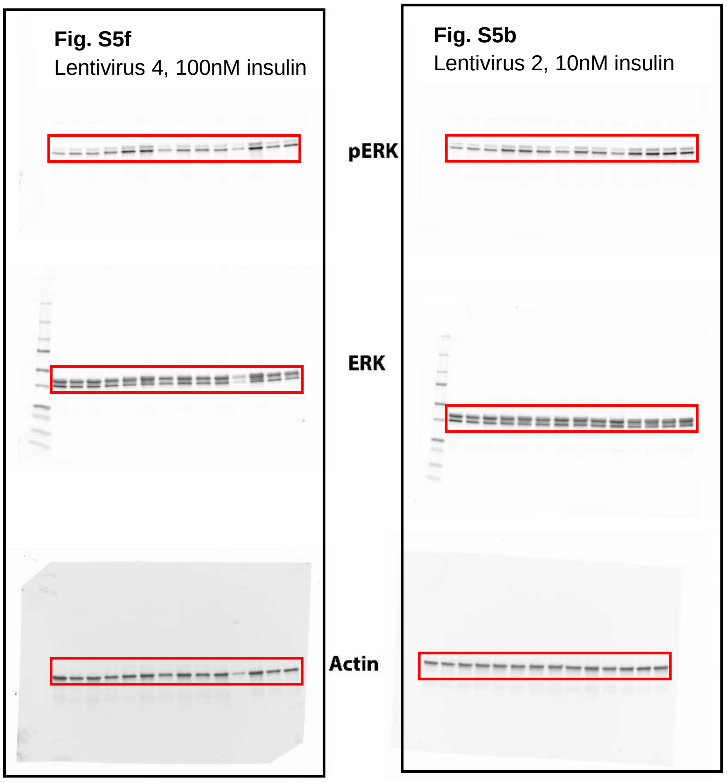

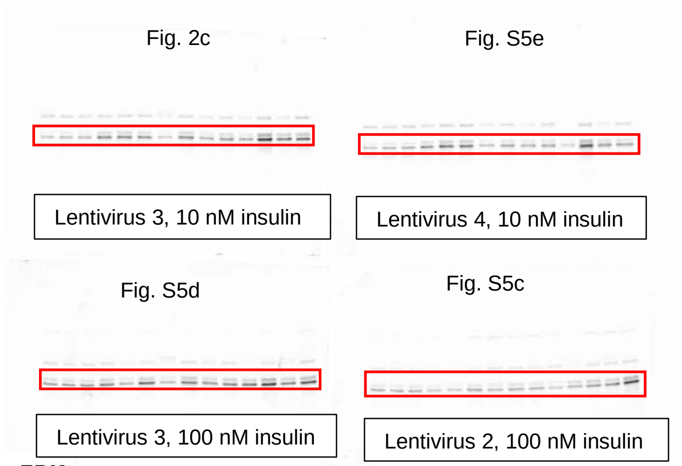

pERK

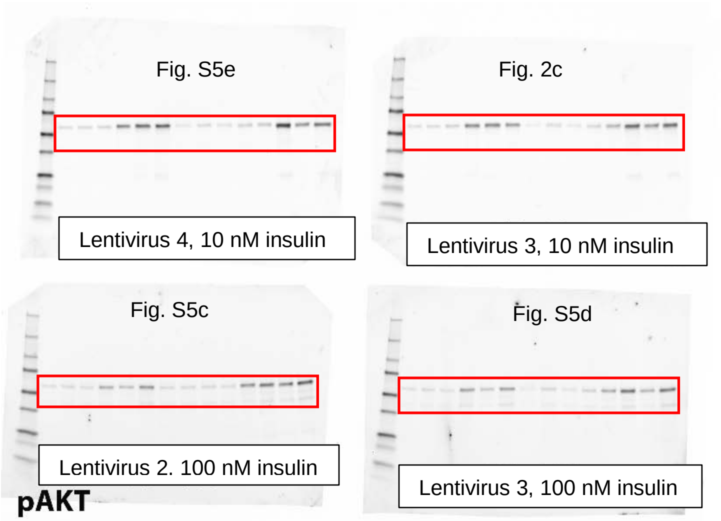

pAKT

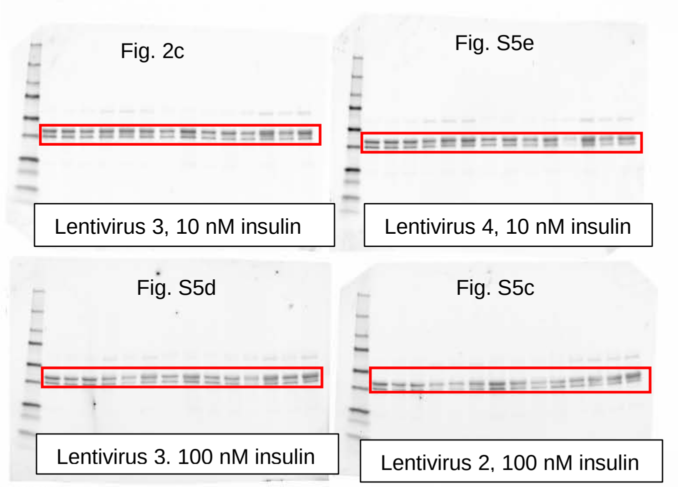

ERK

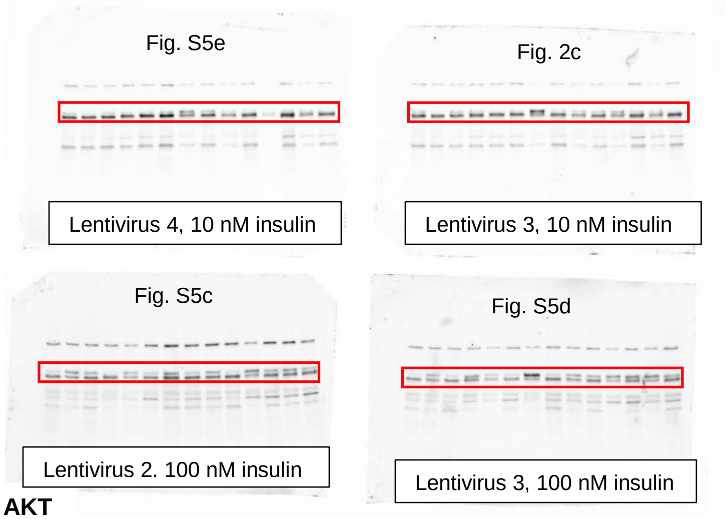

AKT

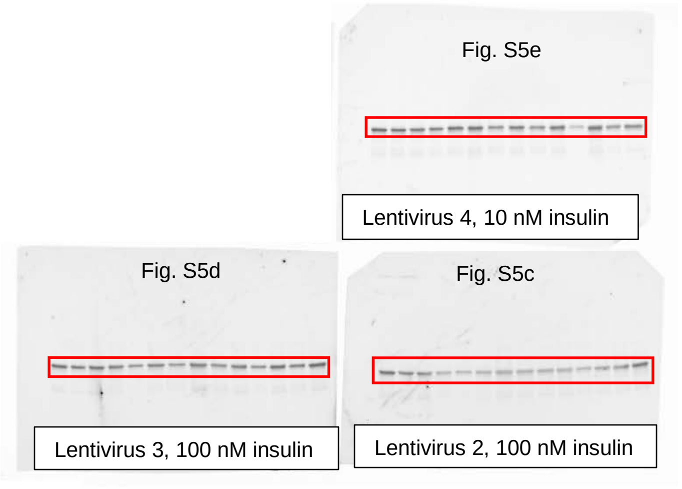

Actin

Blots from Figure 3

High-Fat Diet: Hippocampus

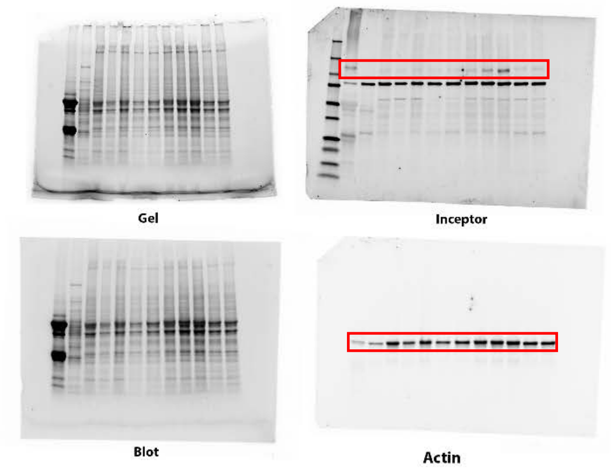

High-Fat Diet: Cortex

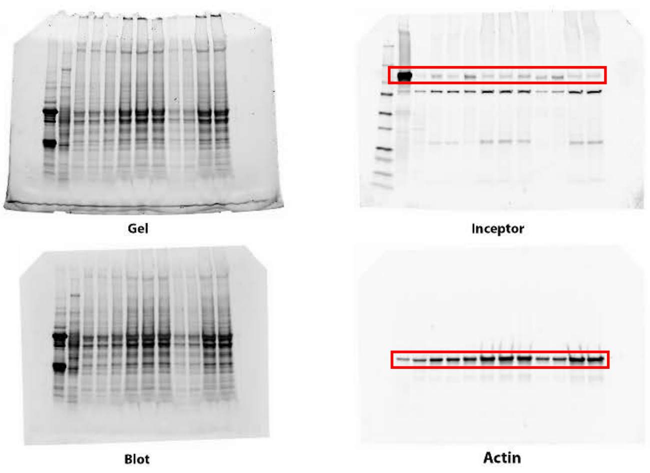

High-Fat Diet: Hypothalamus

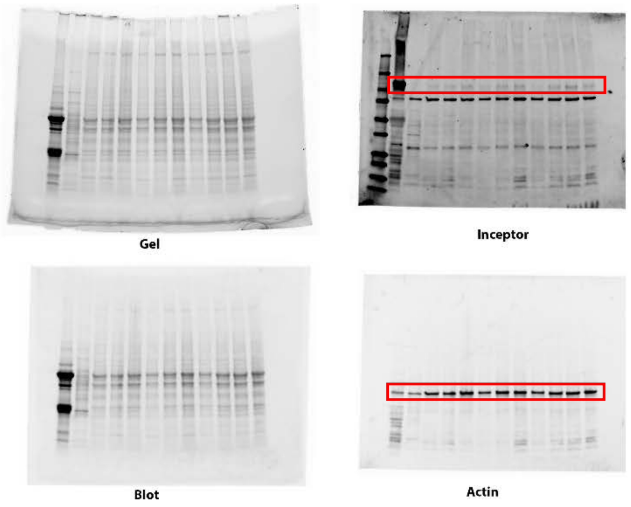

High-Fat Diet: Pancreas

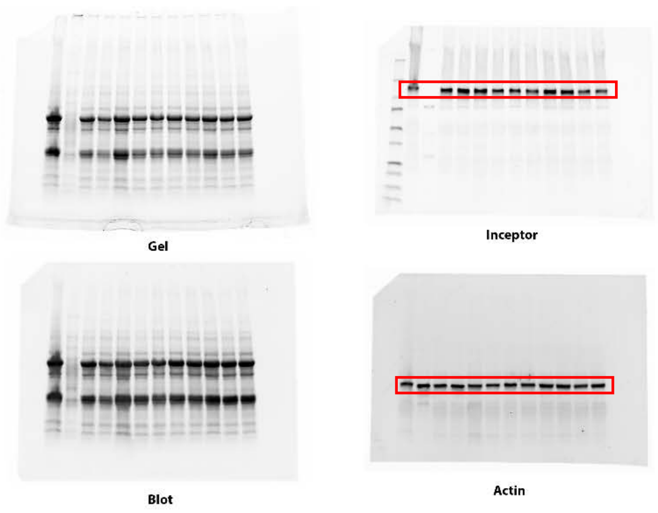

Blots from Figure 4

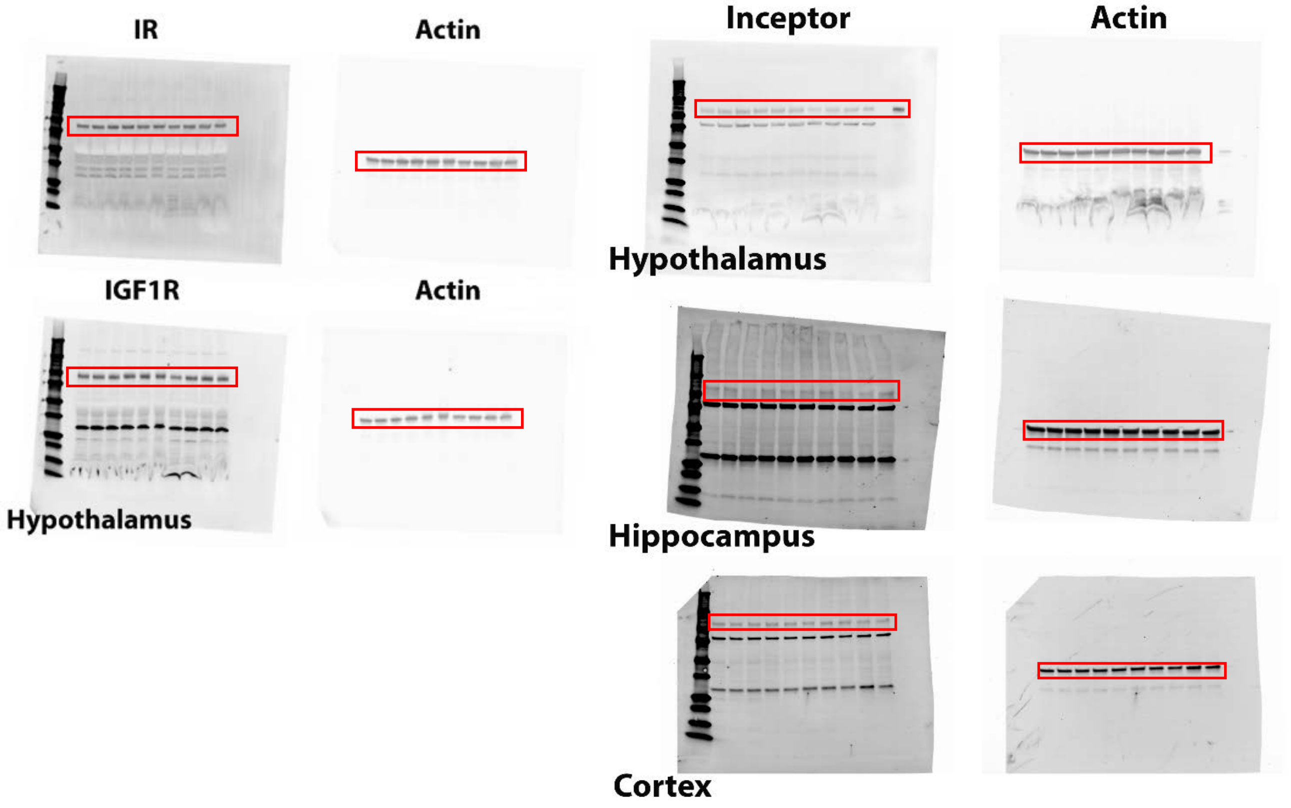

Blots from Figure 5

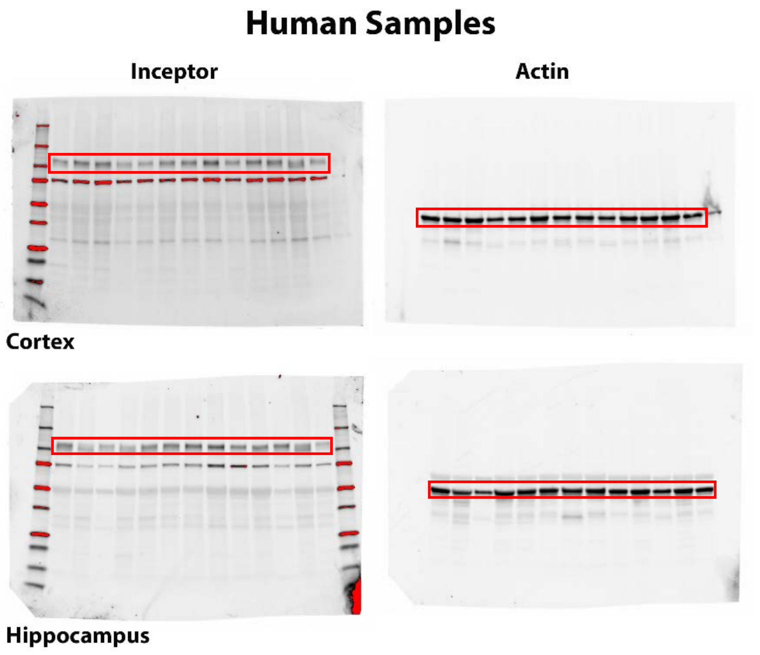

Blots from Figure S4

From Figure S4a:

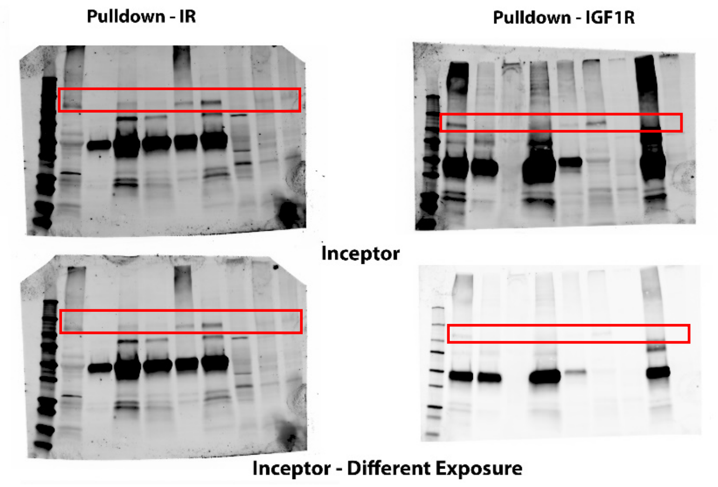

From Figure S4b:

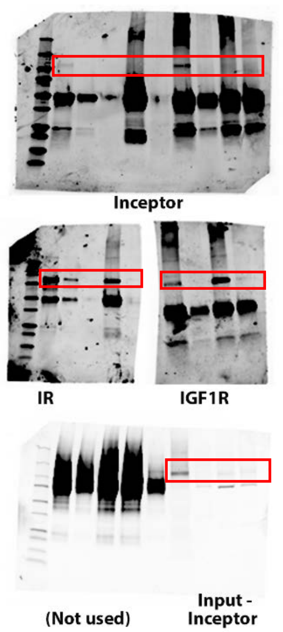

Supplement: Supplementary file 1 — Supplementary Information. [file 41598_2023_36248_MOESM1_ESM.pdf]
